# Comparative efficacy and safety of prostacyclin therapies for pulmonary arterial hypertension: a systematic review and network meta-analysis

**DOI:** 10.3389/fmed.2025.1643220

**Published:** 2025-10-13

**Authors:** Khaled M. Saleh, Jihad Mallat, Samiuddin Mohammed, Govinda Bodi, Hosam Alazazzi, Simi Salim, Mohamed Elhennawi, Talha Iqbal, Hani Sabbour

**Affiliations:** ^1^Respiratory Division, Integrated Hospital Institute, Cleveland Clinic Abu Dhabi, Abu Dhabi, United Arab Emirates; ^2^Critical Care Division, Integrated Hospital Institute, Cleveland Clinic Abu Dhabi, Abu Dhabi, United Arab Emirates; ^3^Cleveland Clinic Lerner College of Medicine, Case Western Reserve University, Cleveland, OH, United States; ^4^Royal College of Surgeons in Ireland – Bahrain, Al Sayh Muharraq Governorate, Adliya, Bahrain; ^5^The Jawaharlal Institute of Postgraduate Medical Education & Research (JIPMER), Puducherry, India; ^6^Medical School of Malaysia, Kuala Lumpur, Malaysia; ^7^Mediclinic Hospitals & Clinics in Abu Dhabi, Abu Dhabi, United Arab Emirates; ^8^Brown University Warren Alpert School of Medicine, Providence, RI, United States

**Keywords:** pulmonary arterial hypertension, prostacyclin therapies, treprostinil, epoprostenol, selexipag, iloprost, beraprost

## Abstract

**Background:**

Pulmonary arterial hypertension (PAH) is a progressive, fatal cardiopulmonary disorder characterized by elevated pulmonary vascular resistance leading to right heart failure. Current treatment utilizes pathway-specific vasodilators, including numerous prostacyclin therapies with diverse delivery methods. Despite available options, head-to-head studies comparing these treatments remain scarce.

**Aim:**

This network meta-analysis seeks to systematically evaluate all prostacyclin-based PAH therapies to guide clinical decision-making regarding treatment selection.

**Methods:**

We implemented a frequentist approach to network meta-analysis (NWM). For continuous outcomes, we calculated pooled mean differences (MD), whereas risk ratios (RR) were determined for binary endpoints. All estimates incorporated 95% confidence intervals. Results achieving *p*-values below 0.05 were considered statistically significant.

**Results:**

Our NWM comprising 32 studies (N = 7,819) revealed significant mortality reduction with treprostinil versus placebo (RR 0.66, 95%CI 0.49–0.90), while epoprostenol transitioned demonstrated superior survival benefit (P-score 0.78). For functional capacity, epoprostenol exhibited the greatest 6-Minute Walking Distance (6MWD) improvement (46.84 m, 95%CI 21.90–71.78; P-score 0.90) versus placebo. Hemodynamically, epoprostenol achieved optimal Pulmonary Arterial Pressure (PAP) reduction (−6.29 mmHg, 95%CI -6.99 to −5.59; P-score 0.95), while iloprost demonstrated superior Pulmonary Vascular Resistance (PVR) improvement (−342.09, 95%CI -410.30 to −273.87; P-score 1.00). Epoprostenol ranked highest for Right Atrial Pressure (RAP) reduction (−2.41 mmHg, 95%CI -2.65 to −2.18) and cardiac index improvement (0.56, 95%CI 0.49–0.63). Regarding clinical worsening, selexipag showed potential superiority (RR 0.62, 95%CI 0.51–0.74; P-score 0.95) compared to treprostinil (P-score 0.55).

**Conclusion:**

Our NMA demonstrates that prostacyclin pathway therapies offer benefits in PAH management. While epoprostenol exhibits superior improvements in hemodynamics and functional capacity, treprostinil reduces mortality by 34%, and selexipag excels in preventing clinical worsening and hospitalizations.

## Introduction

Pulmonary arterial hypertension (PAH) is a progressive and fatal cardiopulmonary disorder marked by elevated pulmonary vascular resistance, leading to right heart failure (1). US claims data (1999–2007) indicate a population prevalence of 109 cases per million, rising to 451 per million in Medicare beneficiaries (2). PAH is clinically defined by a resting mean pulmonary arterial pressure >20 mmHg with pulmonary arterial wedge pressure ≤15 mmHg and pulmonary vascular resistance ≥3 Wood units (3, 4). Pathologically, it reflects an imbalance between vasodilatory (nitric oxide, prostacyclin) and vasoconstrictive (endothelin) pathways (5).

Contemporary PAH management employs several mechanistically distinct vasodilatory agents targeting specific pathophysiological pathways, including endothelin receptor antagonists, phosphodiesterase type 5 inhibitors, soluble guanylate cyclase activators, and prostaglandin pathway modulators (5). This targeted approach addresses the complex vascular pathobiology underlying PAH, potentially improving patient outcomes through pathway-specific intervention rather than generalized vasodilation alone (5).

Treatment selection is guided by clinical and functional indicators associated with disease progression risk (6). Initial intervention typically employs phosphodiesterase type 5 inhibitors and endothelin receptor antagonists—frequently administered in combination—for patients presenting with low-to-intermediate risk profiles (6). Treatment intensification with prostaglandin pathway modulators becomes necessary for suboptimal responders, with complex cases of severe PAH generally warranting combination therapy utilizing two or three agents (6).

The prostacyclin metabolic pathway plays a crucial role in PAH pathophysiology, with patients exhibiting diminished prostacyclin synthase expression and reduced urinary metabolites (7, 8). Therapeutic agents targeting this pathway exert multiple beneficial effects through vasodilation, platelet aggregation inhibition, cytoprotection, and antiproliferative activity (8). Since its 1995 approval, epoprostenol has remained fundamental in managing severe PAH, despite requiring continuous intravenous administration due to its brief half-life (3–5 min). Clinical trials have demonstrated its ability to improve symptoms, exercise capacity, hemodynamics, and mortality in idiopathic and scleroderma-associated PAH (6, 9–11).

The prostacyclin therapeutic arsenal has expanded to include multiple agents with varied administration routes. Inhaled iloprost demonstrated benefits in a controlled trial comparing multiple daily inhalations against placebo (12–15). Treprostinil, available in subcutaneous, intravenous, inhaled, and oral formulations, improved various clinical parameters across multiple studies, though administration-specific challenges exist, including injection-site pain with the subcutaneous route (16–22). Beraprost showed only modest, transient exercise capacity improvements without sustained benefits (23, 24). Selexipag, an orally available selective prostacyclin receptor agonist structurally distinct from prostacyclin, reduced morbidity/mortality risk by 40% in a large phase 3 trial (25).

Despite the availability of multiple prostacyclin-based therapies, direct comparative studies evaluating the relative efficacy and safety of all these agents are notably lacking. This evidence gap complicates clinical decision-making regarding optimal agent selection, administration route, and sequential therapy strategies. Our network meta-analysis aims to address this critical knowledge gap by comprehensively comparing all prostacyclin treatments in PAH.

## Methods

This study adhered to Preferred Reporting Items for Systematic Reviews and Meta-Analyses-network meta-analysis (PRISMA-NMA) methodology and conformed to the established protocols described in the Cochrane Handbook for Systematic Reviews of Interventions (26, 27).

### Literature search

A systematic literature retrieval was implemented across multiple electronic repositories (PubMed, Cochrane Central, Scopus, and Web of Science) encompassing all indexed publications from database inception through April 2025. The search strategy employed structured terminology combinations as detailed in Supplementary Table 1. This electronic search was supplemented by manual examination of previous systematic reviews and reference lists from eligible studies to ensure comprehensive identification of relevant research.

### Eligibility criteria

Our systematic review and NWM encompassed studies examining prostacyclin pathway analogues (treprostinil, iloprost, selexipag, epoprostenol, or beraprost) in subjects with hemodynamically-confirmed pulmonary hypertension. Diagnostic verification required right heart catheterization demonstrating characteristic parameters: MPAP ≥25 mmHg, PCWP ≤15 mmHg, and PVR exceeding three Wood units. Pharmacological interventions were considered irrespective of administration route, or therapeutic duration. Comparison arms encompassed these therapeutic agents with one another, placebo interventions, or standard treatment approaches. Methodological parameters restricted analysis to complete, peer-evaluated, English-language publications, thereby excluding experimental studies, conference abstracts without corresponding full manuscripts, and non-published research findings.

Our key primary endpoints were as follows: all-cause mortality, six-minute walk distance (6MWD), hemodynamic parameters (pulmonary arterial pressure, pulmonary vascular resistance, right atrial pressure, cardiac index), and clinical deterioration as defined in the primary studies.

### Data extraction

Two independent reviewers systematically extracted data from eligible trials into standardized databases, resolving disagreements through consensus discussion. Extraction parameters encompassed comprehensive study characteristics (identification, design, population size, geographical location), intervention details (dosage, administration route, follow-up duration), demographic information (age distribution, gender representation), disease classification (idiopathic/hereditary versus other PH etiologies), functional assessments (6-min walk distance, mean pulmonary arterial pressure), symptom severity (NYHA functional classification II-IV), concurrent pharmacotherapy (calcium channel blockers, digoxin), enrollment criteria, and principal conclusions.

### Quality assessment

Two independent reviewers conducted quality assessments using the Cochrane Risk of Bias Tools for Randomized Studies (ROB 2) tool for randomized trials, Nonrandomized Clinical Studies (ROBINS-I) for non-randomized studies, and the Newcastle-Ottawa Scale (NOS) for observational studies. The ROB 2 tool assessed five bias domains: randomization process, intervention adherence, missing data handling, outcome measurement, and selective reporting. Each domain was rated as “low risk,” “some concerns,” or “high risk,” contributing to an overall study rating (28). For non-randomized studies, ROBINS-I evaluated seven domains mirroring a pragmatic RCT framework: confounding, participant selection, intervention classification, protocol deviations, missing data, outcome measurement, and selective reporting. Studies received overall ratings of “low,” “moderate,” “serious,” “critical” risk, or “no information” (29). The NOS appraised observational studies across three domains: participant selection, group comparability, and outcome ascertainment (30).

The certainty of the evidence for key outcomes was evaluated using the Grading of Recommendations, Assessment, Development and Evaluations (GRADE) framework, adapted for network meta-analyses (31, 32). The initial certainty for each outcome, derived from a network of randomized trials, was considered ‘High’. Downgrading was considered across five domains: risk of bias, inconsistency, indirectness, imprecision, and publication bias (31, 32). To provide a comprehensive assessment, we separately rated the certainty of direct evidence (from head-to-head trials), indirect evidence (evaluating for intransitivity), and the final network estimate (evaluating for incoherence). This process was conducted by two independent reviewers, and the final ratings and detailed rationale are presented in a Summary of Findings table.

### Data analysis

We performed a frequentist network meta-analysis to synthesize outcome data and estimate treatment effects. For continuous outcomes, we calculated mean differences (MDs) with 95% CIs, while risk ratios (RRs) with 95% CIs were used for dichotomous outcomes, considering *p* < 0.05 statistically significant. For each outcome, we generated network plots, forest plots (placebo-referenced), and league tables summarizing all direct and indirect comparisons. Treatment ranking was assessed using P-scores, which represent the probability that a treatment is better than another, averaged over all competing treatments, with values closer to 1.0 indicating superior efficacy. Analyses used a random-effects model (R *netmeta* package), with heterogeneity assessed via *I^2^* and chi-square tests (*X^2^-p*); values of *I^2^* ≥ 50% or *X^2^-p* < 0.05 indicated substantial heterogeneity (33). Meta-regression was not feasible due to limitations in the available data and the lack of consistent stratification of effect modifiers across outcomes in the included studies.

## Results

### Literature search

**Figure 1:**
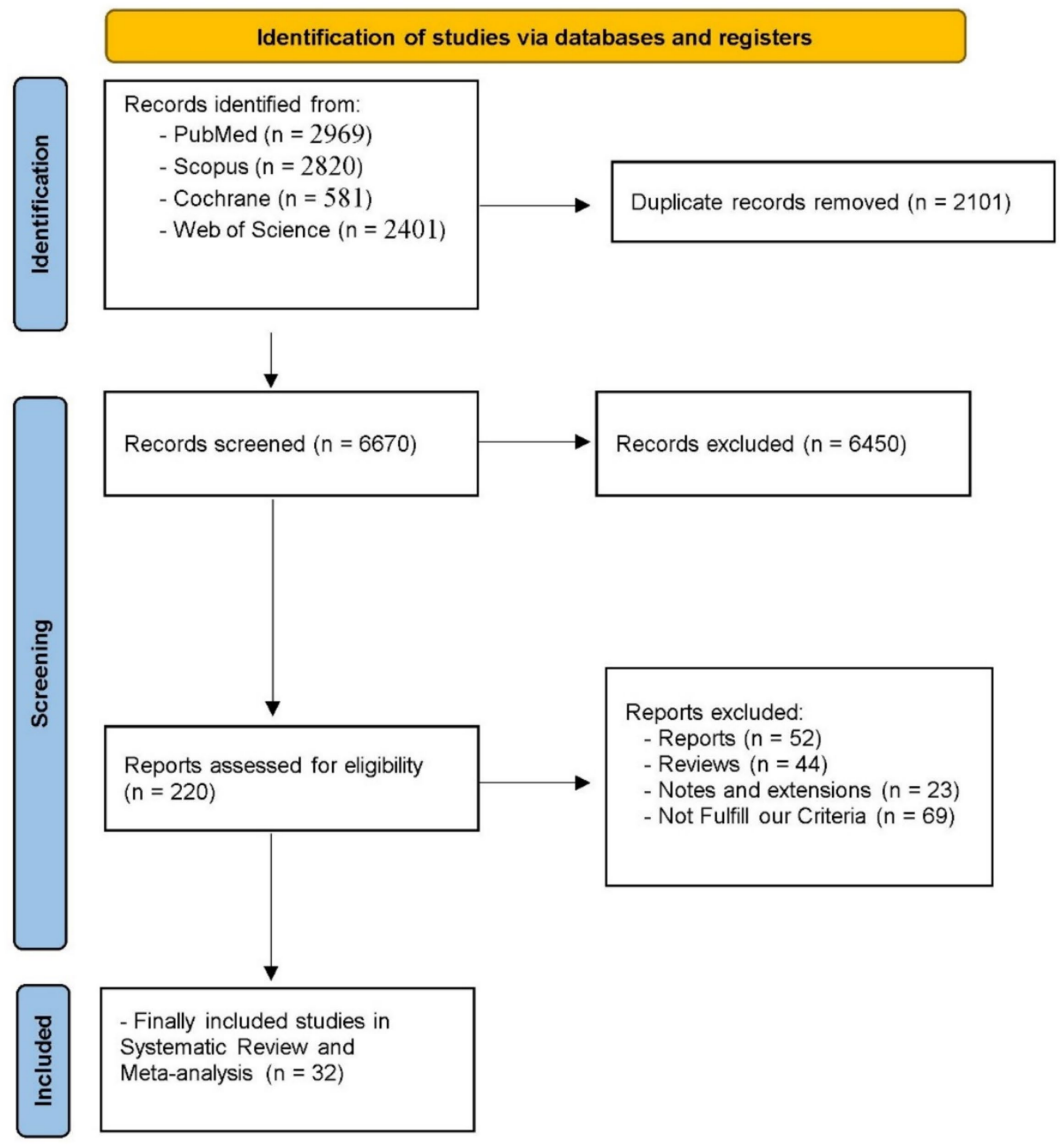
PRISMA flow diagram of the studies selection process.

Our systematic search yielded 8,771 records from four databases: PubMed (*n* = 2,969), Scopus (*n* = 2,820), Cochrane (*n* = 581), and Web of Science (*n* = 2,401). After removing 2,101 duplicates, 6,670 unique records were screened for relevance. Of these, 6,450 were excluded based on title and abstract review, leaving 220 full-text articles for comprehensive eligibility assessment. A further 188 reports were excluded for specific reasons: general reports (*n* = 52), reviews (*n* = 44), notes and extensions (*n* = 23), and studies not fulfilling our predetermined inclusion criteria (*n* = 69). Ultimately, 32 studies met all eligibility criteria and were included in our systematic review and network meta-analysis (9, 12–25, 34–50). This rigorous selection process is summarized in the PRISMA flow diagram in Figure 1.

### Included studies characteristics

A total of 32 studies were included in this meta-analysis, comprising 24 randomised controlled trials, three open-label trials, and five cohort studies, with a cumulative sample size of 7,819 patients. The studies had follow-up periods ranging from one to 48 months. Five prostacyclin analogues were evaluated: treprostinil (*n* = 16 studies), iloprost (*n* = 5), selexipag (*n* = 6), epoprostenol (*n* = 4), and beraprost (*n* = 3). The mean age of participants across studies ranged from 32 to 68.3 years, with female predominance (41.7–91%). The majority of studies included patients with idiopathic or hereditary pulmonary hypertension (43.1–97%), while others incorporated various PAH etiologies, including connective tissue disease-associated, HIV-associated, and congenital heart disease-associated PAH. Baseline 6-min walk distance, where reported, ranged from 231.4 to 445 meters, and mean pulmonary arterial pressure ranged from 35.2 to 66 mmHg. Most studies enrolled patients with WHO/NYHA functional class II-IV, with a predominance of class III patients. Key inclusion criteria typically specified PAH diagnosis confirmed by right heart catheterizations (MPAP≥25 mmHg, PCWP ≤15 mmHg, PVR > 3 Wood units) and baseline exercise capacity limitations. Table 1 shows baseline characteristics and the summary of the included studies.

**Table 1:** Baseline characteristics and summary of the included studies.

Study ID	Arms	Sample size	Study design	Country	Dose	Follow up (m)	Route	Age, mean (SD)	Female, *n* (%)	Idiopathic or hereditary PH, *n* (%)	Other PH, *n* (%)	6MWD (m), mean (SD)	PAPm (mm Hg), mean (SD)	NYAH II, *n* (%)	NYAH III, *n* (%)	NYAH IV, *n* (%)	Calcium channel blockers, *n* (%)	Digoxin, *n* (%)	Inclusion criteria	Conclusion
Barst 2003 (23)	Beraprost	60	RCTs	US	Starting at 20 μg four times daily (QID), titrated up to a median dose of 120 μg	12	Oral	42 (2)	52 (87)	47 (78)	13 (22)	433 (11)	56 (2)	NA	NA	NA	NA	NA	Age >8 years, WHO class II or III PAH (PPH, collagen vascular disease-related, or congenital shunts), Baseline peak VO₂: 8–28 mL/kg/min, Mean pulmonary artery pressure ≥25 mm Hg, pulmonary capillary wedge pressure ≤15 mm Hg, pulmonary vascular resistance >3 U.	These data suggest that beneficial effects may occur during early phases of treatment with beraprost in WHO functional class II or III patients but that this effect attenuates with time.
	Placebo	56						42 (2)	47 (84)	39 (70)	17 (30)	445 (10)	55 (2)	NA	NA	NA	NA	NA		
Barst 1996 (9)	Epoprostenol	41	RCTs	US and Canada	Initial dose: 4 ng/kg/min, Titrated to mean final dose of 9.2 ng/kg/min	3	IV	40 (3)	31 (76)	NA	NA	316 (18)	61 (2)	NA	31 (76)	10 (24)	NA	NA	Diagnosis of PPH, NYHA functional class III or IV despite optimal conventional therapy, On stable regimens of anticoagulants, oral vasodilators, diuretics, cardiac glycosides, or supplemental oxygen.	As compared with conventional therapy, the continuous intravenous infusion of epoprostenol produced symptomatic and hemodynamic improvement, as well as improved survival in patients with severe primary pulmonary hypertension.
	Conventional therapy	40					NA	40 (2)	28 (70)	NA	NA	272 (23)	59 (2)	NA	29 (73)	11 (28)	NA	NA		
Burger 2023 (35)	Treprostinil	405	Cohort study	US	NA	12	Inhalation	65.5 (13.5)	272 (67.2)	NA	NA	NA	NA	NA	NA	NA	NA	NA	Adults ≥18 years old, ≥1 inpatient or ≥2 outpatient PAH diagnoses (ICD-9: 416.0/8/9; ICD-10: I27.0/2/8/9), Continuous health plan enrollment 6 months pre-index and 12 months post-index, No prior treprostinil/iloprost use in pre-index period.	The results suggest that inhaled treprostinil is less burdensome, is associated with greater adherence and persistence, and may reduce all-cause hospitalizations and ED visits.
	Iloprost	62					Inhalation	64.0 (13.2)	43 (69.4)	NA	NA	NA	NA	NA	NA	NA	NA	NA		
Frantz 2015 (36)	Epoprostenol naïve	147	Cohort study	US	2.0 ng/kg/min	12	IV	50 (14.6)	258 (76.8)	NA	NA	308.2 (113.4)	54.8 (13.1)	NA	NA	NA	NA	NA	Diagnosed with PAH (group 1 pulmonary hypertension), Parenteral-naive or parenteral-transitioned (from another epoprostenol or treprostinil formulation to RTS-Epo).	Risk of hospitalization and mortality remain high in patients with PAH. In particular, patients who are parenteral-naive at initiation of RTS-Epo therapy, male patients, and patients with CRI require close monitoring and aggressive clinical management.
	Epoprostenol transitioned	189			29.0 ng/kg/min		IV			NA	NA	350.5 (133.8)	51.4 (15.1)	NA	NA	NA	NA	NA		
Galiè 2003 (7)	Beraprost	65	RCTs	Multicentric	80 μg four times daily; range up to 120 μg	3	Oral	45.8 (16.3)	42 (64.6)	35 (53.9)	30 (46.1)	362 (94)	NA	31 (47.7)	34 (52.3)	NA	17 (26.2)	12 (18.5)	Age >8 years, NYHA class II/III PAH (primary or associated with collagen disease, congenital shunts, portal hypertension, or HIV), Baseline 6MWD 50–500 m, mPAP >25 mmHg, PCWP <15 mmHg.	Beraprost improves exercise capacity and symptoms in NYHA functional class II and III patients with PAH and, in particular, in those with primary pulmonary hypertension.
	Placebo	65			111 μg four times daily			45.1 (14.4)	38 (58.5)	28 (43.1)	37 (56.9)	383 (93)	NA	33 (50.8)	32 (49.2)	NA	11 (16.9)	13 (20.0)		
Gessler 2017 (37)	Iloprost BREELIB nebulizer	27	RCTs	Multicentric	5 μg	30	Inhalation	58 (16)	21 (78)	NA	NA	NA	NA	NA	NA	NA	NA	NA	Confirmed PAH (mPAP >25 mmHg, PCWP <15 mmHg, PVR > 4 Wood units), WHO functional class III, Stable on inhaled iloprost 5 μg via I-Neb prior to study, Concomitant PAH therapies allowed if stable (≥3 months for ERAs/PDE5 inhibitors).	The BREELIB nebulizer offers reduced inhalation time, good tolerability, and may improve iloprost aerosol therapy convenience and thus compliance for patients with PAH
	Iloprost I-Neb nebulizer	26			5 μg		Inhalation							NA	NA	NA	NA	NA		
Hill 2022 (21)	Treprostinil naïve	66	CTs	US	26.5 mcg	13	Inhalation	55 (14.6)	52 (78.8)	NA	NA	NA	NA	37 (56.1)	29 (43.9)	NA	NA	NA	Age ≥18, WHO Group 1 PAH, Stable doses for ≥3 months of: Nebulized treprostinil (Transition) OR ≤2 non-prostacyclin oral therapies (Naïve), NYHA FC II-IV, 6MWD ≥ 150 m, FEV1 ≥ 60%, FEV1/FVC ≥ 60%	Yutrepia represents a significant advancement in inhaled prostacyclin therapy for PAH, offering enhanced convenience and lifestyle flexibility through its easy-to-use dry-powder inhaler, whether transitioning from nebulized therapy or initiating treatment.
	Treprostinil transitioned	55			26.5–106 mcg		Inhalation	53.3 (14.1)	47 (85.5)	NA	NA	NA	NA	43 (78.2)	12 (21.8)	NA	NA	NA		
Hiremath 2010 (38)	Treprostinil	30	RCTs	India	72 ng/kg/min	3	IV	35.5 (12.3)	19 (63)	29 (97)	1 (3)	259.2 (65)	64 (3)	NA	29 (97)	1 (3)	NA	17 (57)	WHO Group 1 PAH (idiopathic, familial, HIV-associated, or collagen vascular disease-associated), Mean pulmonary arterial pressure >35 mm Hg, Pulmonary capillary wedge pressure <16 mm Hg, Pulmonary vascular resistance >5 mm Hg/liter/min, Age 16–75 years, Stable NYHA Class III or IV symptoms.	We conclude that treprostinil treatment significantly improved exercise capacity, dyspnea and functional class. Several plasma proteins that might track disease were abnormal at baseline, and changes were associated with improved exercise capacity.
	Placebo	14			80 ng/kg/min		IV	39.3 (13)	8 (57)	13 (93)	1 (7)	231.4 (73.7)	66 (6)	NA	13 (93)	1 (7)	NA	9 (64)		
Ismat 2022 (39)	Treprostinil	24	RCTs	US	TPIP 112.5 μg, 225 μg, 450 μg, 675 μg	1	Inhalation	32 (5.6)	10 (41.6)	NA	NA	NA	NA	NA	NA	NA	NA	NA	Healthy adults aged 18–45 years, BMI 19.0–32.0 kg/m^2^, Non-smokers, No clinically significant abnormalities in medical history, labs, or ECG.	TPIP was well tolerated at the doses tested, and dose titration improved tolerability. Treprostinil pharmacokinetics were linear and supportive of a QD treatment regimen. These results support further development of TPIP in patients with PAH and PH-ILD.
	Placebo	2			NA		NA	30 (2.8)	1 (50)	NA	NA	NA	NA	NA	NA	NA	NA	NA		
Jing 2013 (40)	Treprostinil	233	RCTs	Multicentric	3.4 mg	3	Oral	41.5 (10.9)	172 (74)	171 (73)	61 (26)	332.3 (71.6)	NA	NA	NA	NA	NA	NA	12 to 75 years of age, Idiopathic or hereditary PAH (including PAH associated with appetite suppressant/toxin use), PAH associated with repaired congenital systemic-to-pulmonary shunts (repaired ≥2 years), or PAH associated with collagen vascular disease or HIV, Baseline 6MWD between 100 and 450 m.	Oral treprostinil improves exercise capacity in PAH patients not receiving other treatment. Oral treprostinil could provide a convenient, first-line prostacyclin treatment option for PAH patients not requiring more intensive therapy.
	Placebo	116			NA			42.5 (9.9)	90 (78)	88 (76)	28 (24)	325.2 (77.1)	NA	NA	NA	NA	NA	NA		
Khan 2022 (41)	Tre-prostinil	34	RCTs	Multicentric	5.5 mg	6	Oral	44.1 (14.4)	23 (67.6)	NA	NA	NA	NA	NA	NA	NA	NA	NA	Patients with PAH on a stable dose of oral PAH monotherapy at study entry, Participants of the FREEDOM-EV trial who consented to the hemodynamic sub-study.	Oral treprostinil significantly improved pulmonary artery compliance, cardiac output, and reduced pulmonary vascular resistance compared to placebo in PAH patients.
	Placebo	27			NA			40.1 (14.6)	23 (85)	NA	NA	NA	NA	NA	NA	NA	NA	NA		
Klose 2021 (42)	Oral Selexipag	20	Prospective, multi-center, open-label, single-sequence, crossover, phase III study	US and Germany	400–1,600 μg	1	IV	56.5 (9.43)	16 (80)	14 (70)	6 (30)	NA	NA	NA	NA	NA	NA	NA	Adults aged 18–75 years with PAH (WHO FC I–III), Stable oral selexipag dose (no changes in PAH medications/diuretics for ≥28 days), No moderate/severe hepatic impairment, severe renal failure, or uncontrolled hyperthyroidism, Systolic BP ≥ 90 mmHg at screening.	Temporarily switching between corresponding doses of oral and IV selexipag was well-tolerated with no unexpected safety findings and comparable exposure to the active metabolite. Treatment with IV selexipag is a feasible option to bridge temporary oral selexipag treatment interruptions.
	IV Selexipag	20			450–1800 μg		Oral					NA	NA	NA	NA	NA	NA	NA		
Mcconnell 2020 (43)	Selexipag	123	Cohort study	US	NA	7	Oral	59.5 (14.5)	87 (70.7)	NA	NA	NA	NA	NA	NA	NA	NA	NA	Adults ≥18 years with ≥1 PH diagnosis code (ICD-9/10), Continuous health plan enrollment ≥6 months pre-index, First prescription of oral treprostinil or selexipag between 1/2015–9/2017 (index date), No prior use of index drug or switching between drugs during study.	This study suggests that selexipag is associated with lower hospitalization risk and rate than oral treprostinil.
	Tre-prostinil	99			NA		Oral	63 (15.4)	71 (71.7)	NA	NA	NA	NA	NA	NA	NA	NA	NA		
Mclaughlin 2010 (44)	Tre-prostinil	115	RCTs	US	50 μg	3	Inhalation	55 (10.9)	93 (80.8)	64 (56)	51 (44)	346 (63)	NA	NA	112 (97.4)	118 (98.3)	NA	NA	Adults aged 18–75 years with PAH (idiopathic, familial, or associated with collagen vascular disease, HIV, or anorexigen use), NYHA functional class III or IV, Baseline 6MWD: 200–450 m, Stable dose of bosentan (125 mg/day) or sildenafil (≥20 mg tid) for ≥3 months.	This trial demonstrates that, among PAH patients who remain symptomatic on bosentan or sildenafil, inhaled treprostinil improves exercise capacity and quality of life and is safe and well-tolerated.
	Placebo	120			52 μg		Inhalation	52 (11.3)	98 (81.6)	67 (56)	53 (44)	351 (69)	NA	NA	3 (2.6)	2 (1.7)	NA	NA		
Mclaughlin 2002 (11)	Iloprost	34	RCTs	US	26.8 μg	3	Inhalation	51 (14)	27 (79)	17 (50)	17 (50)	331 (64)	51 (11)	0	35 (97)	1 (3)	NA	NA	Age 10–80 years with PAH (idiopathic or associated with collagen vascular disease, HIV, or anorexigen use), Stable bosentan therapy (≥4 months), Baseline 6-MWD: 100–425 m, NYHA functional class II–IV (94% class III), Hemodynamics: mPAP >25 mmHg, PCWP <15 mmHg, PVR ≥ 240 dyn·s·cm^−5^.	Adding inhaled iloprost to bosentan improved exercise capacity (6-MWD), functional class, and hemodynamics, while delaying clinical worsening, Well-tolerated with expected prostanoid-related side effects (e.g., cough, headache).
	Placebo	33			27.8 μg		Inhalation	49 (15)	26 (79)	20 (61)	13 (39)	340 (73)	52 (13)	1 (3)	30 (91)	2 (6)	NA	NA		
Mclaughlin 2003 (16)	Tre-prostinil	17	RCTs	US	13 ng/kg/min	2	SC	37 (17)	21 (81)	NA	NA	373 (25)	59 (4)	NA	25 (96)	1 (4)	NA	NA	Diagnosis of PPH (NIH Registry criteria), NYHA functional class III/IV, Baseline 6-MWD: 50–450 m, Hemodynamics: mPAP ≥25 mmHg, PCWP ≤15 mmHg, PVR > 3 Wood units.	Subcutaneous treprostinil has favorable hemodynamic effects when given acutely and in the short term. Treprostinil can be given safely to an ambulatory patient with a novel subcutaneous delivery pump system.
	Placebo	9			38.9 ng/kg/min		SC	37 (17)		NA	NA	384 (27)	64 (6)	NA			NA	NA		
Ogo 2022 (46)	Selexipag	39	RCTs	Japan	200 μg twice daily, titrated up to a maximum of 1,600 μg twice daily	5	Oral	66.3 (11.1)	29 (74.4)	NA	NA	407.9 (90.9)	35.2 (5.4)	NA	NA	NA	NA	NA	Age 20–85 years, Confirmed CTEPH (by pulmonary ventilation/perfusion scan, angiography, or CT), Haemodynamic criteria:(Mean pulmonary arterial pressure (mPAP) ≥ 25 mmHg, Pulmonary artery wedge pressure (PAWP) ≤ 15 mmHg, PVR > 360 dyn·s·cm^−5^), Inoperable due to distal organised thrombus, high risk (comorbidities/age), or refusal of surgery.	Selexipag significantly improved PVR (−98.2 vs. −4.6 dyn·s·cm^−5^, *p* = 0.006) and other haemodynamic parameters (e.g., cardiac index, Borg score) but did not improve 6MWD or WHO functional class. Adverse events (e.g., headache, diarrhoea) were common but manageable.
	Placebo	39						68.3 (9.6)	29 (74.4)	NA	NA	384 (87)	35.5 (8.3)	NA	NA	NA	NA	NA		
Olschewski 2002 (13)	Iloprost	101	RCTs	Multicentric	30 μg/day	3	Inhalation	51.2 (13.2)	69 (68.3)	51 (50.5)	50 (49.5)	332 (93)	52.8 (11.5)	NA	60 (59.4)	41 (40.6)	NA	NA	Diagnosis of PAH (primary or secondary to appetite suppressants/scleroderma) or inoperable CTEPH, Mean pulmonary artery pressure >30 mmHg, NYHA class III or IV, 6-min walk distance: 50–500 m.	Inhaled iloprost is an effective therapy for patients with severe pulmonary hypertension.
	Placebo	102					Inhalation	52.8 (12)	68 (66.7)	51 (50)	51 (50)	315 (96)	53.8 (14.1)	NA	59 (57.8)	43 (42.2)	NA	NA		
Olschewsk 2010 (14)	Iloprost	30	RCTs	Germany	25 μg/day	24	Inhalation	42 (2.2)	23 (76.7)	20 (66.7)	10 (33.3)	353 (23.5)	57.2 (15.1)	11 (36.7)	13 (43.3)	6 (20.0)	12 (40)	NA	Age 18–70 years, Mean pulmonary arterial pressure (mPAP) ≥ 40 mmHg at rest, Diagnosis: IPAH (idiopathic/familial), PHother (connective tissue disease, chronic thromboembolic PH, interstitial lung disease, etc.).	Inhaled iloprost is well tolerated as long-term therapy and no substantial dose increase is required. Although uncontrolled, the data suggest a long-term clinical benefit from continued therapy with inhaled iloprost.
	Control	33					Inhalation	48.8 (2.1)	21 (63.6)	20 (60.6)	10 (39.4)	330 (22.9)	54.1 (13)	10 (30.3)	17 (51.5)	6 (18.2)	18 (54.5)	NA		
Saji 2016 (15)	Iloprost	68	CTs	North America and Western Europe	NA	3	Inhalation	48.7 (14.1)	45 (66)	51 (75)	17 (25)	336 (98)	NA	NA	40 (59)	28 (41)	NA	NA	Treatment-naïve PAH patients in NYHA FC III/IV.	Inhaled iloprost appeared effective and safe in Japanese PAH patients, including ERA- and PDE5 I-treated patients, consistent with findings of the AIR PAH subpopulation and previous iloprost studies.
	Placebo	78			NA			51.8 (12)	54 (69)	51 (65)	27 (34)	315 (93.7)	NA	NA	46 (59)	32 (41)	NA	NA		
Simonneau 2012 (48)	Selexipag	33	RCTs	Multicentric	200 μg twice daily	4	Oral	54.8 (16.8)	27 (81.8)	25 (75.7)	8 (24.3)	396.2 (71.4)	54.5 (15.3)	NA	NA	NA	NA	NA	Adults (≥18 years) with symptomatic PAH (idiopathic, heritable, connective tissue disease-associated, anorexigen-induced, or congenital heart disease-associated), Stable background therapy with ERAs and/or PDE5-Is for >12 weeks before screening, Baseline PVR > 400 dyn·s·cm^−5^, Two 6MWD tests of 150–500 m (within ±15% of each other).	Selexipag significantly reduced PVR by 30.3% compared to placebo (*p* = 0.0045) and showed favorable trends in 6MWD and WHO FC, Safety profile was consistent with prostacyclin receptor agonism (e.g., headache, jaw pain), with no systemic hypotension.
	Placebo	10					Oral	53.8 (16.3)	8 (80)	8 (80)	2 (20)	350.3 (123.5)	54.6 (13.8)	NA	NA	NA	NA	NA		
Sitbon 2015 (25)	Selexipag	574	RCTs	Multicentric	200 μg twice daily, up-titrated weekly in 200 μg	17.6	Oral	48.2 (15.19)	457 (79.6)	325 (56.6)	249 (43.4)	358.5 (76.31)	NA	NA	NA	NA	NA	NA	Confirmed PAH (right heart catheterization: PVR ≥ 5 Wood units [400 dyn·s·cm^−5^]), 6MWD: 50–450 m, Stable background therapy (if applicable: ERAs, PDE5-Is, or both for ≥3 months).	Among patients with pulmonary arterial hypertension, the risk of the primary composite end point of death or a complication related to pulmonary arterial hypertension was significantly lower with selexipag than with placebo. There was no significant difference in mortality between the two study groups.
	Placebo	582				16	Oral	47.9 (15.55)	466 (80.1)	350 (60.1)	232 (39.9)	348 (83.23)	NA	NA	NA	NA	NA	NA		
Spikes 2022 (17)	Tre-prostinil 32 μg	2	CTs	US	32 μg	2	Inhalation	48 (28.3)	2 (100)	1 (50)	1 (50)	NA	NA	NA	NA	NA	NA	NA	Adults (≥18 years) diagnosed with PAH (6th World Symposium on Pulmonary Hypertension group 1 PAH), Started treprostinil inhalation solution ≥3 months before the baseline visit and on a stable dosing regimen (no change in dose within 30 days of baseline, 6–12 breaths four times daily).	The transition from treprostinil inhalation solution to TreT is safe, well-tolerated, and accompanied by statistically significant improvements in key clinical assessments and patient-reported outcomes, with comparable systemic exposure between the two formulations at evaluated doses.
	Tre-prostinil 48 μg	27			48 μg		Inhalation	54.7 (13.1)	22 (81.5)	17 (63)	10 (37)	NA	NA	NA	NA	NA	NA	NA		
	Tre-prostinil 64 μg	22			64 μg		Inhalation	58 (12.8)	19 (86.4)	11 (50)	11 (50)	NA	NA	NA	NA	NA	NA	NA		
Tanabe 2020 (49)	Selexipag	25	RCTs	Japan	100 μg twice daily (bid) up to a maximum of 800 μg bid	4	Oral	58 (15)	17 (68)	NA	NA	378 (77)	41.1 (11.7)	13 (52)	11 (44)	1 (4)	NA	NA	Japanese patients aged 20–75 years, Diagnosis of CTEPH confirmed by pulmonary ventilation/perfusion scan and angiography within 2 years, NYHA/WHO functional class II–IV, Baseline hemodynamics (via right heart catheterization):(mPAP ≥25 mmHg, PAWP <15 mmHg, PVR > 400 dyn·s/cm^3^.), Stable anticoagulation for ≥90 days prior to enrollment.	Selexipag treatment improved pulmonary hemodynamics in Japanese patients with CTEPH, but PVR did not show a significant difference between the selexipag and placebo groups.
	Placebo	9					Oral	60 (5)	7 (77.8)	NA	NA	355 (114)	41.6 (8.5)	5 (55.6)	3 (33.3)	1 (11.1)	NA	NA		
Tapson 2012 (18)	Tre-prostinil	174	RCTs	Multicentric	1 mg twice daily (bid), titrated weekly in 1-mg increments	4	Oral	51 (10.9)	148 (85)	113 (65)	61 (35)	346.1 (71.4)	NA	41 (24)	127 (73)	4 (2)	NA	NA	Age 12–70 years with symptomatic PAH (idiopathic, familial, or associated with congenital heart disease, connective tissue disease, or HIV), Mean pulmonary arterial pressure >25 mmHg, pulmonary capillary wedge pressure ≤15 mmHg, PVR > 3 Wood units, Baseline 6MWD: 100–450 m, Stable background ERA and/or PDE-5 inhibitor therapy for ≥90 days (stable dose for ≥30 days).	The primary end point of improvement in 6MWD at week 16 did not achieve significance. This study enhanced understanding of oral treprostinil titration and dosing, which has set the stage for additional studies.
	Placebo	176					Oral	50 (10.58)	140 (80)	119 (68)	55 (32)	345.4 (75.5)	NA	31 (18)	139 (79)	5 (3)	NA	NA		
Tapson 2013 (50)	Tre-prostinil	157	RCTs	Multicentric	Initiated at 0.25 mg twice daily (bid), titrated weekly to a maximum of 12 mg bid	4	Oral	51.5 (12.6)	119 (76)	104 (66)	53 (35)	329.4 (69.2)	NA	43 (27)	110 (71)	3 (2)	NA	NA	Age 18–75 years with idiopathic PAH (IPAH), familial PAH (FPAH), or PAH associated with connective tissue disease, congenital heart disease, or HIV, Stable background therapy (ERA, PDE-5 inhibitor, or both) for ≥90 days (stable dose for ≥30 days), Baseline 6MWD: 150–425 m, Hemodynamic confirmation of PAH (mPAP >25 mmHg, PCWP ≤15 mmHg, PVR > 3 Wood units).	The addition of oral treprostinil to background ERA and PDE-5I therapy did not result in a statistically significant improvement in exercise capacity. Side effects were common but tolerated by most subjects.
	Placebo	153						50.4 (10.39)	122 (80)	99 (65)	54 (35)	336.8 (63.5)	NA	37 (24)	115 (76)	0 (0)	NA	NA		
Waxman 2021 (19)	Tre-prostinil	163	RCTs	US	6 μg per breath; target dose of 9 breaths (54 μg) four times daily	4	Inhalation	65.6 (12)	85 (52.1)	NA	NA	NA	NA	NA	NA	NA	NA	NA	Adults ≥18 years with interstitial lung disease (diagnosed by CT within 6 months), Confirmed group 3 pulmonary hypertension by right heart catheterization (PVR > 3 Wood units, PCWP ≤15 mm Hg, mPAP ≥25 mm Hg), Baseline 6MWD ≥ 100 m.	In patients with pulmonary hypertension due to interstitial lung disease, inhaled treprostinil improved exercise capacity from baseline, assessed with the use of a 6-min walk test, as compared with placebo.
	Placebo	163						67.4 (9.3)	68 (41.7)	NA	NA	NA	NA	NA	NA	NA	NA	NA		
White 2020 (22)	Tre-prostinil	346	RCTs	Multicentric	3.56 mg TID	48	Oral	45.66 (15.7)	275 (79.5)	219 (63.3)	127 (36.7)	392.96 (92.5)	NA	NA	NA	NA	NA	NA	Age 18–75 years, WHO Group 1 PAH confirmed by right heart catheterization (mPAP ≥25 mmHg, PCWP ≤15 mmHg), Stable oral monotherapy (PDE5 inhibitor, ERA, or riociguat) for ≥30 days, Baseline 6MWD ≥ 150 m.	Adding oral treprostinil to background monotherapy significantly reduced the risk of clinical worsening in PAH patients, with improvements in NT-proBNP, functional class, and dyspnea. Safety profile was consistent with prostacyclin-class therapy (high rates of headache, diarrhea).
	Placebo	344						44.86 (15.4)	269 (78.2)	216 (62.8)	128 (37.2)	398.56 (100)	NA	NA	NA	NA	NA	NA		
Zamanian 2016 (20)	Tre-prostinil	666	cohort study	US	NA	19	Inhalation	52.5 (13.6)	519 (78)	341 (51)	350 (52.5)	NA	NA	245 (37)	332 (50)	37 (6)	NA	NA	Clinical diagnosis of WHO Group 1 PAH, Prescribed inhaled treprostinil (intervention) or other FDA-approved PAH therapy (control).	Overall, inhaled treprostinil was well tolerated by PAH patients in routine clinical care, with respiratory-related AEs consistent with the known safety profile.
	Control	667				21.5	Mixed	53.5 (15)	532 (80)	389 (58)	291 (43.6)	NA	NA	329 (49)	239 (36)	24 (4)	NA	NA		
Badesch 2000 (34)	Epoprostenol	56	RCTs	Multicenters	≤2 ng/kg/min	3	IV	53 (13.1)	51 (91)	NA	NA	NA	50.9 (10.6)	NA	NA	NA	NA	NA	Diagnosis of scleroderma spectrum (diffuse/limited scleroderma, CREST, or overlap syndrome), Mean pulmonary artery pressure ≥35 mm Hg, pulmonary vascular resistance ≥3 mm Hg/L/min, 6-min walk distance ≥50 m at baseline, No significant interstitial lung disease.	Continuous epoprostenol therapy improves exercise capacity and cardiopulmonary hemodynamics in patients with pulmonary hypertension due to the scleroderma spectrum of disease.
	Conventional therapy	55			NA	3	Mixed	57.3 (10.3)	45 (82)	NA	NA	NA	49.1 (10.2)	NA	NA	NA	NA	NA		
Nagaya 1999 (45)	Bera-prost sodium	24	cohort study	Japan	60–180 μg/day	30	Oral	39 (18)	15 (62.5)	NA	NA	NA	7 (3)	NA	22 (91.6)	2 (8.4)	12 (50)	6 (25)	Consecutive PPH patients discharged after first diagnostic catheterization (1981–1997), PPH defined by NIH registry criteria (unexplained pulmonary hypertension).	The oral administration of BPS may have beneficial effects on the survival of outpatients with PPH as compared with conventional therapy alone.
	Conventional therapy	34			NA	44	Oral	33 (13)	23 (67.6)	NA	NA	NA	6 (4)	NA	31 (91.2)	3 (8.8)	13 (38)	24 (70)		
Rubin 1990 (47)	Epoprostenol	11	RCTs	US	7.9 ng/kg·min	2	IV	37.5 (12.1)	7 (64)	NA	NA	NA	NA	NA	NA	NA	NA	1 (9)	Diagnosis of primary pulmonary hypertension (NIH Registry criteria), Stable medication regimen for ≥2 weeks before study.	Prostacyclin produces substantial and sustained hemodynamic and symptomatic responses in severe primary pulmonary hypertension and may be useful in the management of some patients with this disease.
	Conventional therapy	12			NA		Oral	35 (14.8)	9 (75)	NA	NA	NA	NA	NA	NA	NA	NA	3 (25)		

Rcts, Randomized Controlled Trials; Cts, Clinical Trials; M, Months; SD, Standard Deviation; N (%), Number (Percentage); 6MWD, 6-Minute Walking Distance; Papm/Mpap, Mean Pulmonary Arterial Pressure; BMI, Body Mass Index; PH, Pulmonary Hypertension; PAH, Pulmonary Arterial Hypertension; PPH, Primary Pulmonary Hypertension; IPAH, Idiopathic PAH; FPAH, Familial PAH; CTEPH, Chronic Thromboembolic PH; PH-ILD, PH With Interstitial Lung Disease; NYHA/NYAH, New York Heart Association; WHO, World Health Organization; FC, Functional Class; QID, Four Times Daily; TID/Tid, Three Times Daily; Bid, Twice Daily; QD, Once Daily; IV, Intravenous; SC, Subcutaneous; VO₂, Oxygen Consumption; PVR, Pulmonary Vascular Resistance; PCWP/PAWP, Pulmonary Capillary Wedge Pressure; FEV1, Forced Expiratory Volume In 1 Second; FVC, Forced Vital Capacity; ERA, Endothelin Receptor Antagonist; PDE5/PDE-5I, Phosphodiesterase-5 Inhibitor; NA, Not Available; US, United States; ED, Emergency Department.

### Quality assessment

Our systematic quality evaluation encompassed 24 randomized controlled trials (RCTs), 3 non-randomized studies, and 5 cohort studies. Among the RCTs, 17 studies (71%) demonstrated low overall risk of bias, six studies (25%) showed some concerns, and one study (14) exhibited high risk of bias primarily due to deviations from intended interventions. Domain-specific assessment revealed that bias in the randomization process (D1) was most frequent, with 11 RCTs showing concerns. For non-randomized studies, we utilized a 7-domain assessment tool, with only one study (42) achieving low overall risk of bias, while Hill (21) demonstrated serious risk in confounding (D1). The remaining non-randomized studies exhibited a moderate overall risk. All five cohort studies were evaluated with the Newcastle-Ottawa Scale and consistently demonstrated high methodological quality across selection, comparability, and outcome domains. Specifically, all cohort studies received positive ratings for representativeness, exposure ascertainment, outcome assessment, and adequacy of follow-up, with 2 studies (20, 36) achieving the highest scores in cohort comparability. A summary of the quality assessment is presented in the Supplementary Figures 1, 2 and Supplementary Table 1.

The overall certainty of evidence, as assessed by the GRADE framework, ranged from Moderate to Low for most primary and secondary outcomes. A universal one-level downgrade for serious risk of bias was applied across all outcomes due to methodological limitations in a portion of the included studies. Certainty was further downgraded to ‘Low’ for specific outcomes due to additional concerns. Furthermore, the evidence for most head-to-head comparisons between active therapies was downgraded due to imprecision, as confidence intervals were wide and often included the possibility of no effect. A detailed breakdown of the GRADE assessment for all key comparisons is presented in Table 2.

**Table 2:** GRADE summary of findings: certainty of network, direct and indirect estimates for key outcomes and intervention.

Outcome and comparison	Certainty of direct evidence (GRADE)	Certainty of indirect evidence (GRADE)	Network effect estimate (95% CI)	Certainty of network estimate (GRADE)	Detailed rationale for final network rating
Primary outcomes					
All-cause mortality					
*Treprostinil vs. Placebo*	Moderate (Downgraded for risk of bias).	N/A	RR 0.66 (0.49 to 0.90)	Moderate	Downgraded −1 for Risk of Bias: Based on methodological limitations in the included studies.
*Epoprostenol vs. Conventional Rx*	Low (Downgraded for risk of bias and serious imprecision).	N/A	RR 0.28 (0.09 to 0.86)	Low	Downgraded −1 for Risk of Bias and -1 for Imprecision: The CI is very wide, indicating significant uncertainty.
*Epoprostenol vs. Treprostinil*	Low (Downgraded for risk of bias and imprecision).	Moderate (Based on indirect path via placebo).	RR 0.72 (0.45 to 1.15)	Low	Downgraded −1 for Risk of Bias and -1 for Imprecision: The final network CI is wide and crosses the line of no effect, indicating no certainty of a difference between the two agents.
6-Minute walk distance (6MWD)					
*Epoprostenol vs. Placebo*	Low (Downgraded for risk of bias and serious inconsistency).	N/A	MD 46.84 m (21.90 to 71.78)	Low	Downgraded −1 for Risk of Bias and -1 for Inconsistency: The network for this outcome has very serious statistical heterogeneity (I^2^ = 69.7%).
*Iloprost vs. Placebo*	Low (Downgraded for risk of bias and serious inconsistency).	N/A	MD 32.45 m (13.61 to 51.28)	Low	Downgraded −1 for Risk of Bias and -1 for Inconsistency: Inherits the very serious heterogeneity from the overall network.
*Epoprostenol vs. Selexipag*	N/A	Low (Certainty is limited by the low-certainty evidence for Epoprostenol vs. Placebo due to inconsistency).	MD 47.55 m (11.34 to 83.76)	Low	Downgraded −1 for Risk of Bias and -1 for Inconsistency: The estimate is entirely indirect and inherits the very serious inconsistency from the broader network.
Pulmonary arterial pressure (PAP)					
*Epoprostenol vs. Placebo*	Moderate (Downgraded for risk of bias).	N/A	MD -6.29 mmHg (−6.99 to −5.59)	Moderate	Downgraded −1 for Risk of Bias: Based on the overall quality of studies in the network.
*Iloprost vs. Placebo*	Moderate (Downgraded for risk of bias).	N/A	MD -5.56 mmHg (−7.54 to −3.58)	Moderate	Downgraded −1 for Risk of Bias.
*Epoprostenol vs. Treprostinil*	Low (Downgraded for risk of bias and serious imprecision).	Moderate (Based on indirect path via placebo).	MD -8.05 mmHg (−9.96 to −6.15)	Moderate	Downgraded −1 for Risk of Bias. The final network CI is reasonably precise and the estimate is consistent across sources.
Pulmonary vascular resistance (PVR)					
*Iloprost vs. Placebo*	Moderate (Downgraded for risk of bias).	N/A	MD -342.09 (−410.30 to −273.87)	Moderate	Downgraded −1 for Risk of Bias. Evidence for this outcome was homogenous.
*Treprostinil vs. Placebo*	Moderate (Downgraded for risk of bias).	N/A	MD -138.50 (−265.29 to −11.71)	Moderate	Downgraded −1 for Risk of Bias.
*Iloprost vs. Treprostinil*	Moderate (Downgraded for risk of bias).	Moderate (Based on indirect path via placebo).	MD -203.59 (−347.57 to −59.61)	Moderate	Downgraded −1 for Risk of Bias.
Right atrial pressure (RAP)					
*Epoprostenol vs. Placebo*	Moderate (Downgraded for risk of bias).	N/A	MD -2.41 mmHg (−2.65 to −2.18)	Moderate	Downgraded −1 for Risk of Bias.
*Iloprost vs. Placebo*	Moderate (Downgraded for risk of bias).	N/A	MD -2.20 mmHg (−3.49 to −0.91)	Moderate	Downgraded −1 for Risk of Bias.
*Epoprostenol vs. Treprostinil*	Moderate (Downgraded for risk of bias).	Moderate (Based on indirect path via placebo).	MD -3.43 mmHg (−4.25 to −2.61)	Moderate	Downgraded −1 for Risk of Bias. Evidence for this outcome was homogenous and the CI is precise.
Cardiac index					
*Epoprostenol vs. Placebo*	Moderate (Downgraded for risk of bias).	N/A	MD 0.56 (0.49 to 0.63)	Moderate	Downgraded −1 for Risk of Bias.
*Selexipag vs. Placebo*	Moderate (Downgraded for risk of bias).	N/A	MD 0.49 (0.29 to 0.69)	Moderate	Downgraded −1 for Risk of Bias.
*Epoprostenol vs. Selexipag*	N/A	Moderate (Based on indirect path via placebo with no suspicion of intransitivity).	MD 0.07 (−0.17 to 0.31)	Low	Downgraded −1 for Risk of Bias and -1 for Imprecision: The estimate is entirely indirect. The final network CI is wide and crosses zero, indicating no certain difference.
Clinical worsening					
*Selexipag vs. Placebo*	Moderate (Downgraded for risk of bias).	N/A	RR 0.62 (0.51 to 0.74)	Moderate	Downgraded −1 for Risk of Bias.
*Treprostinil vs. Placebo*	Moderate (Downgraded for risk of bias).	N/A	RR 0.73 (0.61 to 0.86)	Moderate	Downgraded −1 for Risk of Bias.
*Selexipag vs. Treprostinil*	Low (Downgraded for risk of bias and serious imprecision).	Moderate (Based on indirect path via placebo).	RR 0.85 (0.66 to 1.10)	Low	Downgraded −1 for Risk of Bias and -1 for Imprecision: The final network CI is wide and crosses the line of no effect, providing no certainty of a difference.
Secondary outcomes					
Hospitalization					
*Selexipag vs. Placebo*	Moderate (Downgraded for risk of bias).	N/A	RR 0.70 (0.55 to 0.89)	Moderate	Downgraded −1 for Risk of Bias.
*Selexipag vs. Treprostinil*	N/A	Moderate (Based on indirect path via placebo with no suspicion of intransitivity).	RR 0.57 (0.40 to 0.80)	Moderate	Downgraded −1 for Risk of Bias. The estimate is entirely indirect but is based on moderate-certainty evidence.
*Selexipag vs. Iloprost*	N/A	Moderate (Based on indirect path via placebo with no suspicion of intransitivity).	RR 0.60 (0.37 to 0.98)	Moderate	Downgraded −1 for Risk of Bias.
NT-proBNP					
*Treprostinil vs. Placebo*	Low (Downgraded for risk of bias and serious imprecision).	N/A	MD -877.17 (−1854.14 to 99.81)	Low	Downgraded −1 for Risk of Bias and -1 for Imprecision: The CI is extremely wide and crosses the line of no effect, indicating profound uncertainty.
*Selexipag vs. Placebo*	Low (Downgraded for risk of bias and serious imprecision).	N/A	MD + 23.10 (−904.64 to 950.84)	Low	Downgraded −1 for Risk of Bias and -1 for Imprecision: The CI is extremely wide and crosses the line of no effect.
*Treprostinil vs. Selexipag*	N/A	Low (Certainty of the indirect path is limited by the low-certainty, imprecise evidence of its components).	MD -900.27 (−2247.55 to 447.02)	Low	Downgraded −1 for Risk of Bias and -1 for Imprecision: The estimate is entirely indirect and the final CI is extremely wide, providing no certainty of a difference.
Adverse events					
Headache					
*Epoprostenol vs. Placebo*	Low (Downgraded for risk of bias and serious imprecision).	N/A	RR 2.51 (1.20 to 5.26)	Low	Downgraded −1 for Risk of Bias and -1 for Imprecision: Due to the very wide CI.
*Selexipag vs. Placebo*	Moderate (Downgraded for risk of bias).	N/A	RR 1.97 (1.38 to 2.79)	Moderate	Downgraded −1 for Risk of Bias.
*Iloprost vs. Beraprost*	Moderate (Downgraded for risk of bias).	Moderate (Based on indirect path via placebo).	RR 0.51 (0.28 to 0.94)	Moderate	Downgraded −1 for Risk of Bias.
Nausea					
*Epoprostenol vs. Placebo*	Low (Downgraded for risk of bias and serious imprecision).	N/A	RR 2.51 (1.20 to 5.26)	Low	Downgraded −1 for Risk of Bias and -1 for Imprecision: Due to the very wide CI.
*Selexipag vs. Placebo*	Moderate (Downgraded for risk of bias).	N/A	RR 1.97 (1.38 to 2.79)	Moderate	Downgraded −1 for Risk of Bias.
*Treprostinil vs. Placebo*	Moderate (Downgraded for risk of bias).	N/A	RR 1.69 (1.40 to 2.05)	Moderate	Downgraded −1 for Risk of Bias.
Diarrhea					
*Selexipag vs. Placebo*	Moderate (Downgraded for risk of bias).	N/A	RR 2.43 (1.58 to 3.73)	Moderate	Downgraded −1 for Risk of Bias.
*Treprostinil vs. Placebo*	Moderate (Downgraded for risk of bias).	N/A	RR 2.19 (1.72 to 2.79)	Moderate	Downgraded −1 for Risk of Bias.
*Selexipag vs. Treprostinil*	N/A	Moderate (Based on indirect path via placebo).	RR 1.11 (0.76 to 1.62)	Low	Downgraded −1 for Risk of Bias and -1 for Imprecision: The estimate is entirely indirect and the final CI is wide and crosses the line of no effect.

### Outcomes

#### All-cause mortality

**Figure 2:**
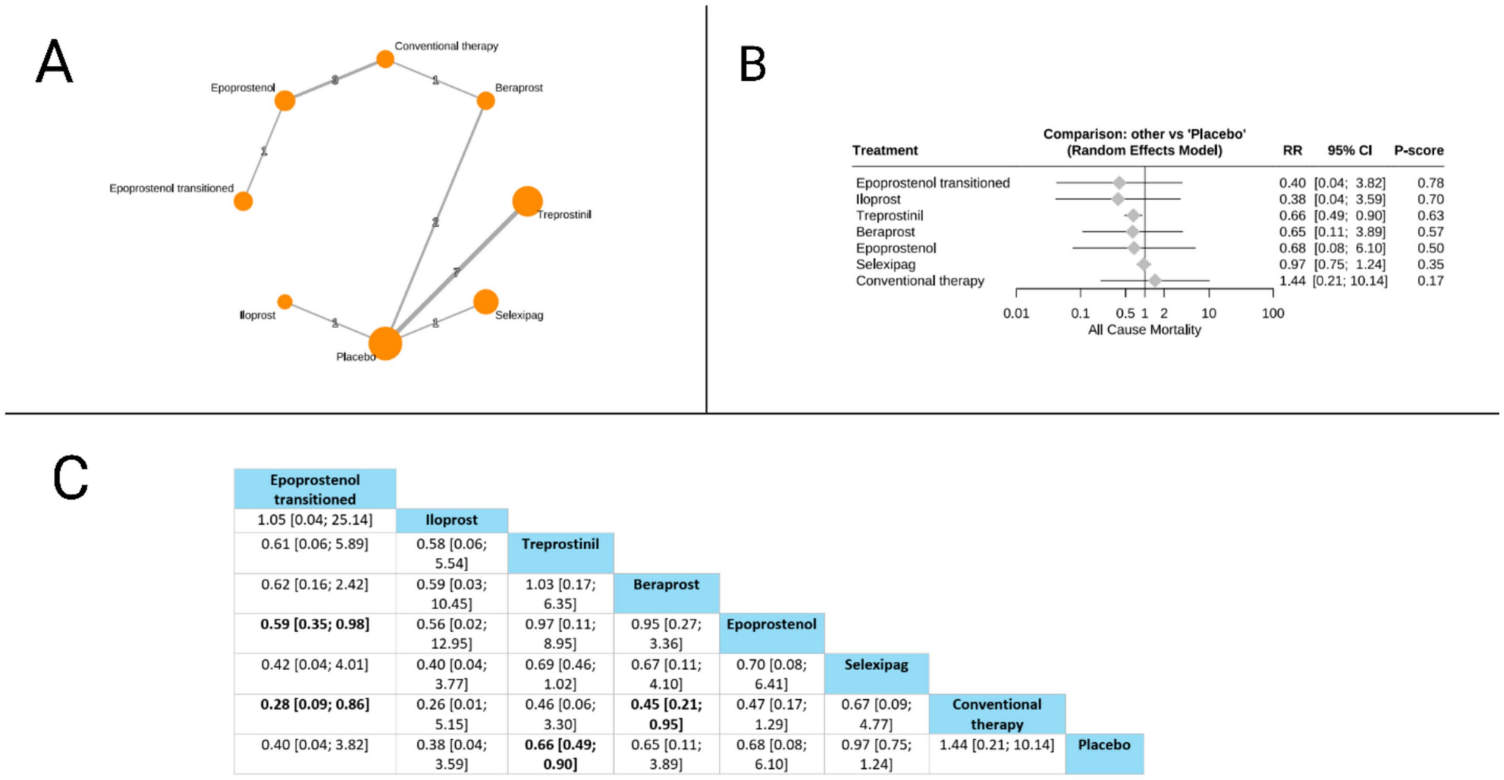
Frequentist random effect model network meta-analysis comparing all-cause mortality of prostanoid therapies, showing **(A)** treatment connections in network plot, **(B)** forest plot of relative risks versus placebo, and **(C)** net league matrix of pairwise comparisons with 95% confidence intervals.

Sixteen studies evaluated the all-cause mortality outcome. Treprostinil demonstrated a statistically significant reduction in all-cause mortality compared to placebo (RR = 0.66, 95% CI: 0.49 to 0.90). When comparing treatments directly, epoprostenol transitioned showed significant mortality benefits over conventional therapy (RR = 0.28, 95% CI: 0.09 to 0.86) and over epoprostenol (RR = 0.59, 95% CI: 0.35 to 0.98). Additionally, beraprost demonstrated superiority over conventional therapy (RR = 0.45, 95% CI: 0.21 to 0.95). According to the P-score ranking, which indicates the probability of each treatment being the best option, epoprostenol transitioned ranked highest (P-score = 0.78), followed by iloprost (P-score = 0.70), treprostinil (P-score = 0.63), beraprost (P-score = 0.57), epoprostenol (P-score = 0.50), selexipag (P-score = 0.35), and conventional therapy (P-score = 0.17). The pooled studies were homogenous with I^2^ = 0% and *X^2^-p* = 0.61 (Figure 2).

#### 6-min walk distance (6MWD)

**Figure 3:**
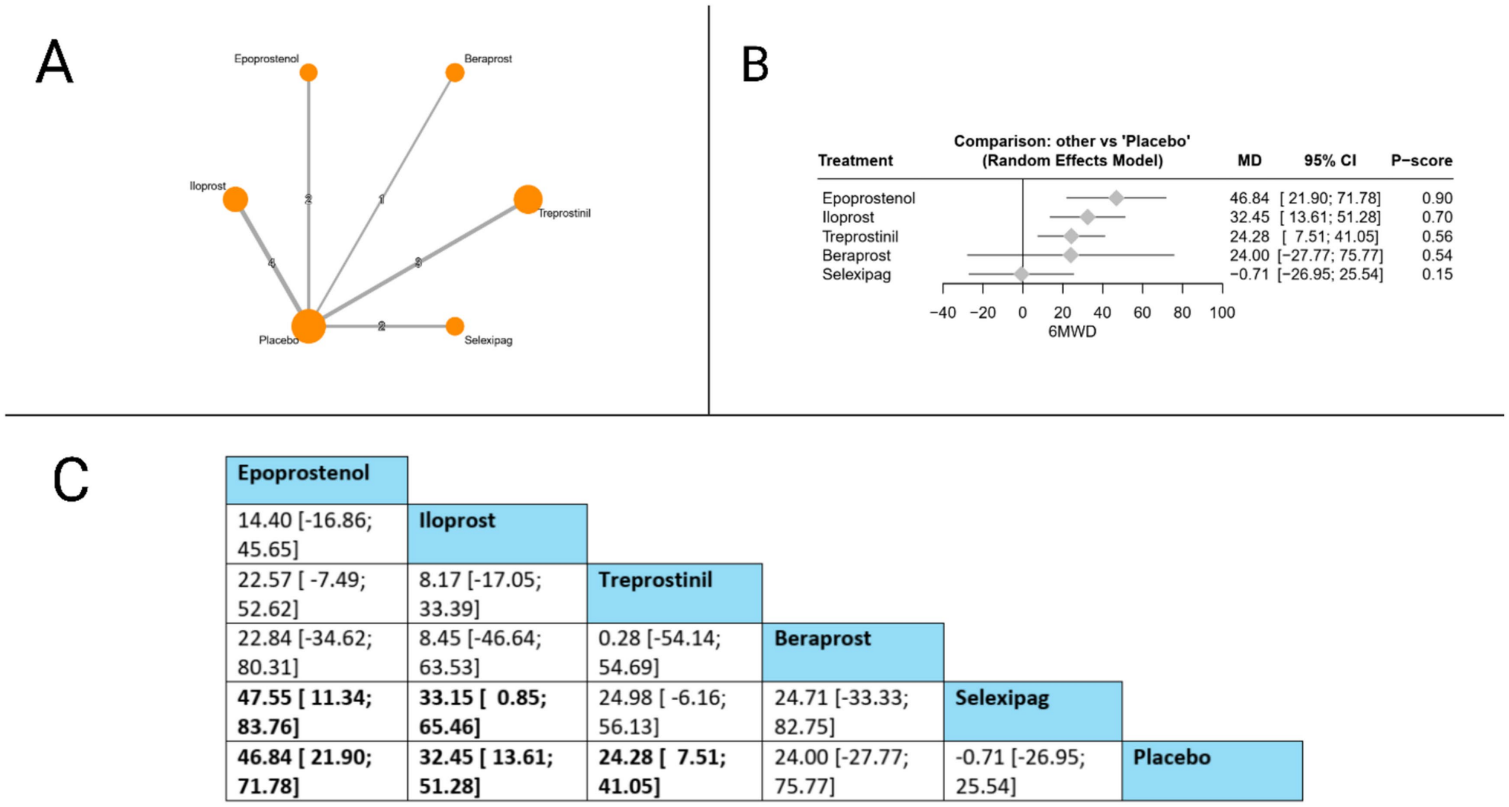
Frequentist random effect model network meta-analysis comparing 6MWD of prostanoid therapies, showing **(A)** treatment connections in network plot, **(B)** forest plot of relative risks versus placebo, and **(C)** net league matrix of pairwise comparisons with 95% confidence intervals.

The 6MWD was evaluated in twelve studies. The forest plot revealed that compared to placebo, three treatments showed statistically significant improvements: epoprostenol (MD = 46.84 meters, 95% CI: 21.90 to 71.78), iloprost (MD = 32.45 meters, 95% CI: 13.61 to 51.28), and treprostinil (MD 24.28 meters, 95% CI: 7.51 to 41.05). The league table further identified significant differences between active treatments, with epoprostenol demonstrating superiority over selexipag (MD = 47.55 meters, 95% CI: 11.34 to 83.76), and iloprost also showing significant benefits over selexipag (MD = 33.15 meters, 95% CI: 0.85 to 65.46). Based on P-scores, which reflect the probability of each treatment being the most effective, epoprostenol ranked highest (P-score = 0.90), followed by iloprost (P-score = 0.70), treprostinil (P-score = 0.56), beraprost (P-score = 0.54), and selexipag (0.15). The pooled studies were heterogeneous with I^2^ = 69.7% and *X^2^-p* = 0.002 (Figure 3).

#### Mean pulmonary artery pressure (MPAP)

**Figure 4:**
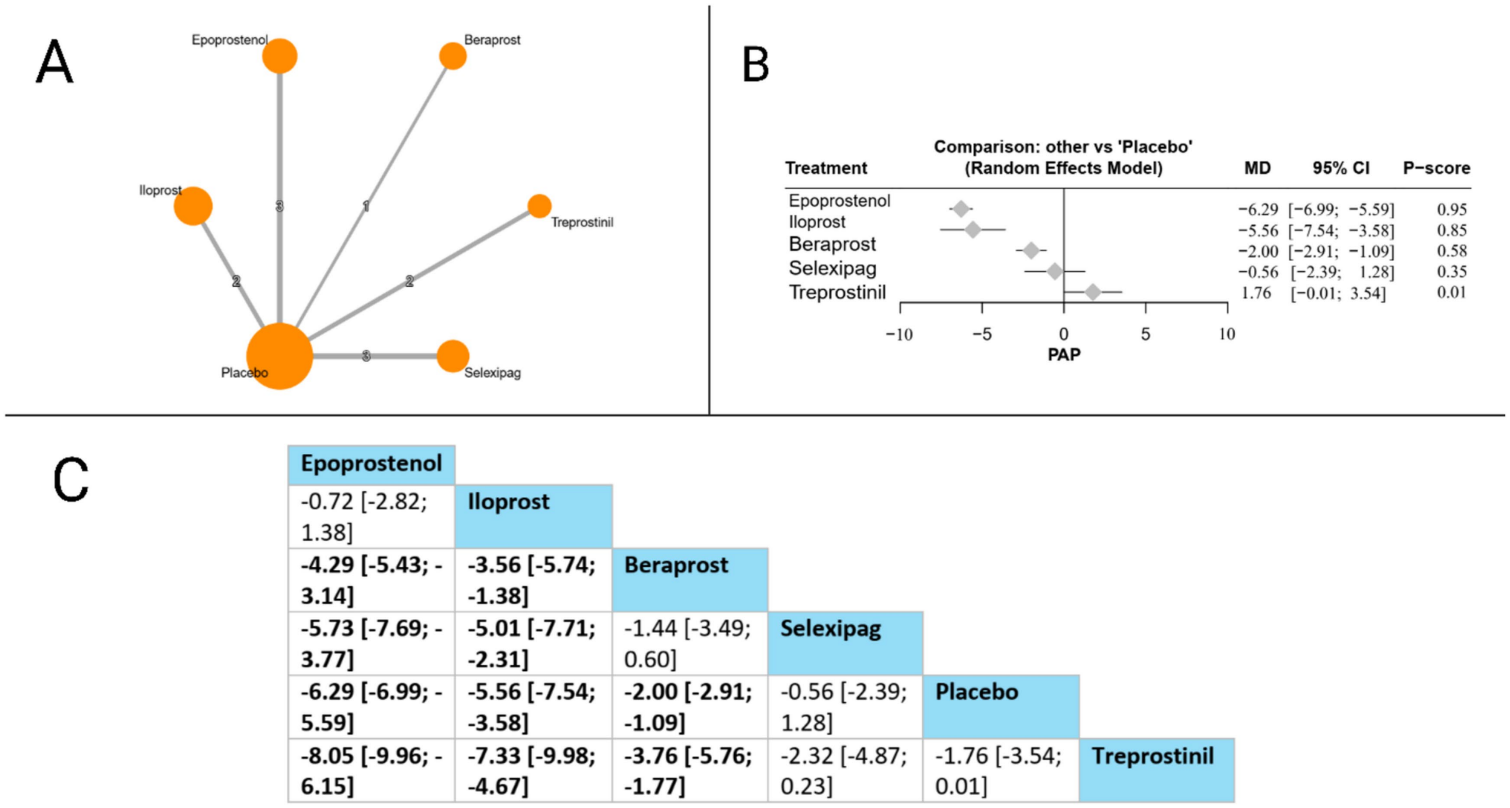
Frequentist random effect model network meta-analysis comparing MPAP mortality of prostanoid therapies, showing **(A)** treatment connections in network plot, **(B)** forest plot of relative risks versus placebo, and **(C)** net league matrix of pairwise comparisons with 95% confidence intervals.

MPAP was evaluated in 11 studies. The forest plot revealed that compared to placebo, three treatments significantly reduced MPAP: epoprostenol (MD = −6.29 mmHg, 95% CI: −6.99 to −5.59), iloprost (MD = −5.56 mmHg, 95% CI: −7.54 to −3.58), and beraprost (MD = −2.00 mmHg, 95% CI: −2.91 to −1.09). In head-to-head comparisons, epoprostenol demonstrated superiority over beraprost (MD = −4.29 mmHg, 95% CI: −5.43 to −3.14), selexipag (MD = −5.73 mmHg, 95% CI: −7.69 to −3.77), and treprostinil (MD = −8.05 mmHg, 95% CI: −9.96 to −6.15). Iloprost also showed significant benefits over beraprost (MD = −3.56 mmHg, 95% CI: −5.74 to −1.38), selexipag (MD = −5.01 mmHg, 95% CI: −7.71 to −2.31), and treprostinil (MD = −7.33 mmHg, 95% CI: −9.98 to −4.67). Additionally, beraprost demonstrated superiority over treprostinil (MD = −3.76 mmHg, 95% CI: −5.76 to −1.77). Based on P-scores, epoprostenol ranked highest (P-score = 0.95), followed by iloprost (P-score = 0.85), beraprost (P-score = 0.58), selexipag (P-score = 0.35), and treprostinil (P-score = 0.01), indicating that epoprostenol offers the most effective MPAP reduction among the evaluated prostacyclin pathway-targeting therapies. The pooled studies were homogenous with *I^2^* = 30.7% and *X^2^-p* = 0.19 (Figure 4).

#### Pulmonary vascular resistance (PVR)

**Figure 5:**
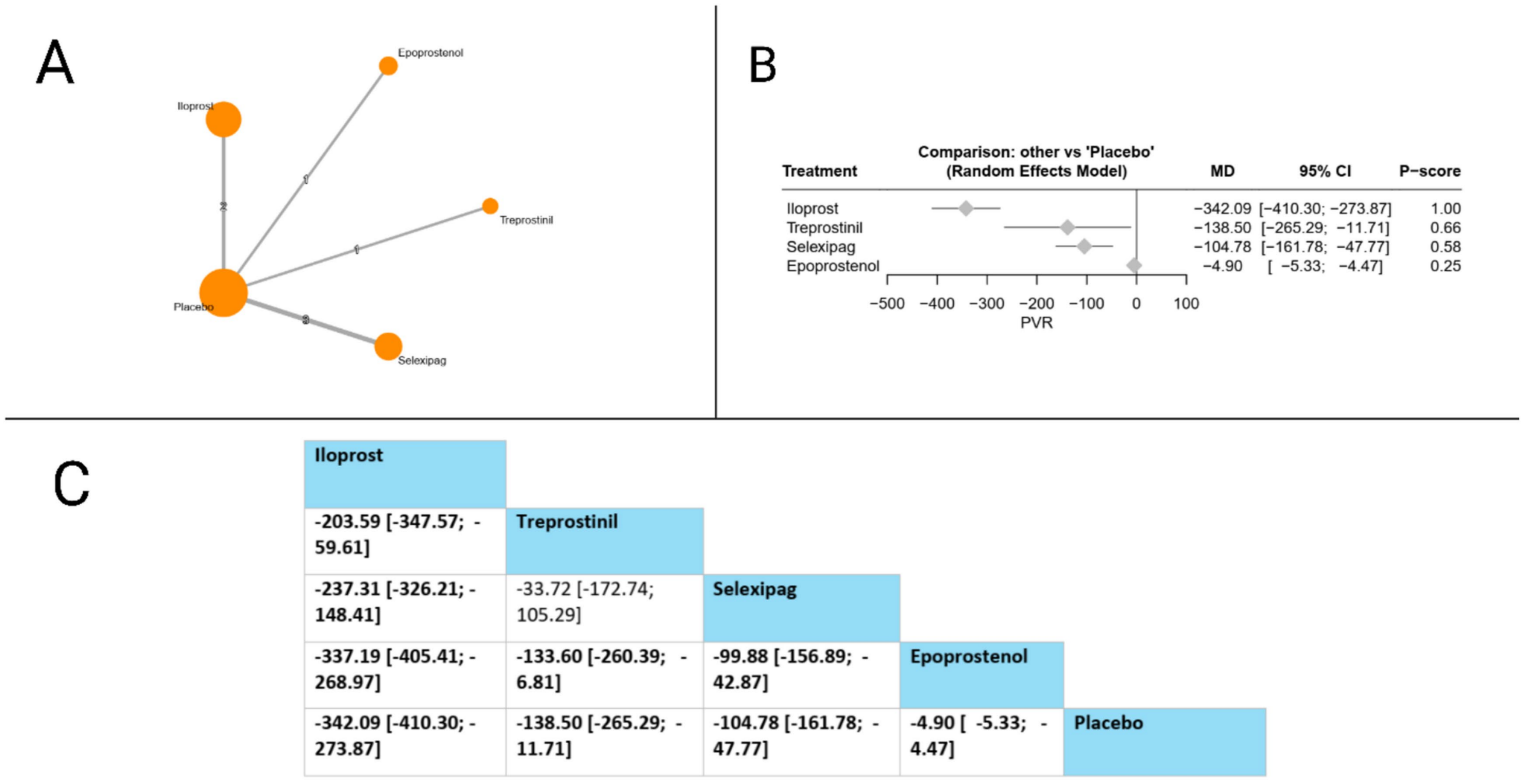
Frequentist random effect model network meta-analysis comparing PVR of prostanoid therapies, showing **(A)** treatment connections in network plot, **(B)** forest plot of relative risks versus placebo, and **(C)** net league matrix of pairwise comparisons with 95% confidence intervals.

PVR was assessed in seven studies. Compared to placebo, all evaluated treatments showed statistically significant PVR reductions: iloprost (MD = −342.09, 95% CI: −410.30 to −273.87), treprostinil (MD = −138.50, 95% CI: −265.29 to −11.71), selexipag (MD = −104.78, 95% CI: −161.78 to −47.77), and epoprostenol (MD = −4.90, 95% CI: −5.33 to −4.47). In head-to-head comparisons, iloprost demonstrated significant superiority over all other treatments, with marked PVR reductions compared to treprostinil (MD = −203.59, 95% CI: −347.57 to −59.61), selexipag (MD = −237.31, 95% CI: −326.21 to −148.41), and epoprostenol (MD = −337.19, 95% CI: −405.41 to −268.97). Additionally, both treprostinil and selexipag showed significant advantages over epoprostenol (MD = −133.60, 95% CI: −260.39 to −6.81 and MD = −99.88, 95% CI: −156.89 to −42.87, respectively). Based on P-scores, which reflect the probability of each treatment being the most effective, iloprost ranked highest (P-score = 1.00), followed by treprostinil (P-score = 0.66), selexipag (P-score = 0.58), and epoprostenol (P-score = 0.25), indicating that iloprost provides the most effective PVR reduction among the prostacyclin pathway-targeting therapies evaluated. The pooled studies were homogenous with *I^2^* = 0% and *X^2^-p* = 0.55 (Figure 5).

#### Right atrial pressure (RAP)

**Figure 6:**
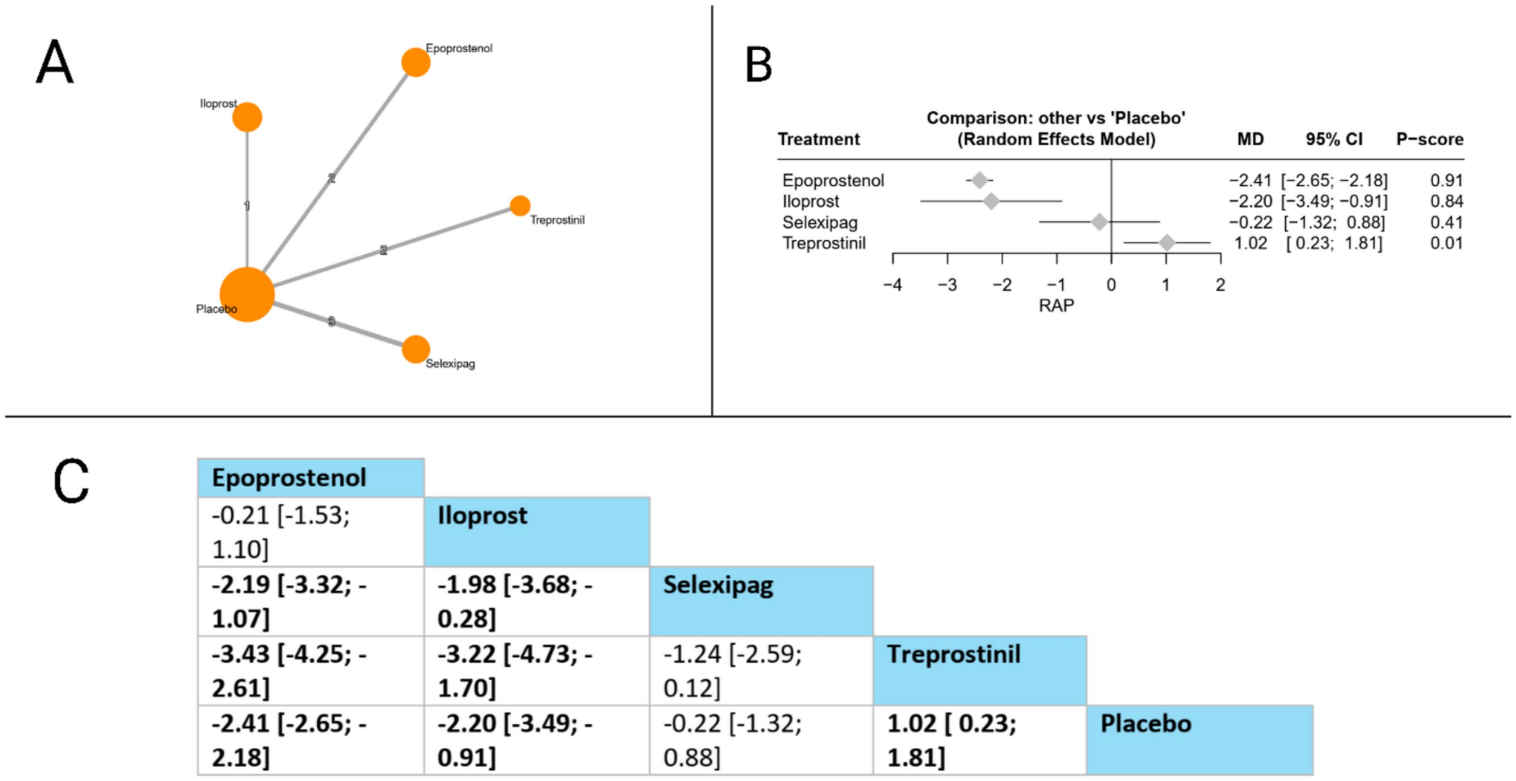
Frequentist random effect model network meta-analysis comparing RAP of prostanoid therapies, showing **(A)** treatment connections in network plot, **(B)** forest plot of relative risks versus placebo, and **(C)** net league matrix of pairwise comparisons with 95% confidence intervals.

Eight studies evaluated RAP. Compared to placebo, epoprostenol (MD = −2.41 mmHg, 95% CI: −2.65 to −2.18) and iloprost (MD = −2.20 mmHg, 95% CI: −3.49 to −0.91) both significantly reduced RAP, while treprostinil surprisingly showed a significant increase in RAP (MD = 1.02 mmHg, 95% CI: 0.23 to 1.81). In head-to-head comparisons, the league table revealed multiple significant differences: epoprostenol was superior to selexipag (MD = −2.19 mmHg, 95% CI: −3.32 to −1.07) and treprostinil (MD = −3.43 mmHg, 95% CI: −4.25 to −2.61); similarly, iloprost demonstrated significant advantages over selexipag (MD = −1.98 mmHg, 95% CI: −3.68 to −0.28) and treprostinil (MD = −3.22 mmHg, 95% CI: −4.73 to −1.70). Based on P-scores, which indicate the probability of each treatment being the most effective, epoprostenol ranked highest (P-score = 0.91), followed by iloprost (0.84), selexipag (P-score = 0.41), and treprostinil ranked lowest (P-score = 0.01). These findings suggest that among prostacyclin pathway-targeting therapies, epoprostenol and iloprost provide the most substantial reductions in right atrial pressure. The pooled studies were homogenous with *I^2^* = 0% and *X^2^-p* = 0.57 (Figure 6).

#### Cardiac index

**Figure 7:**
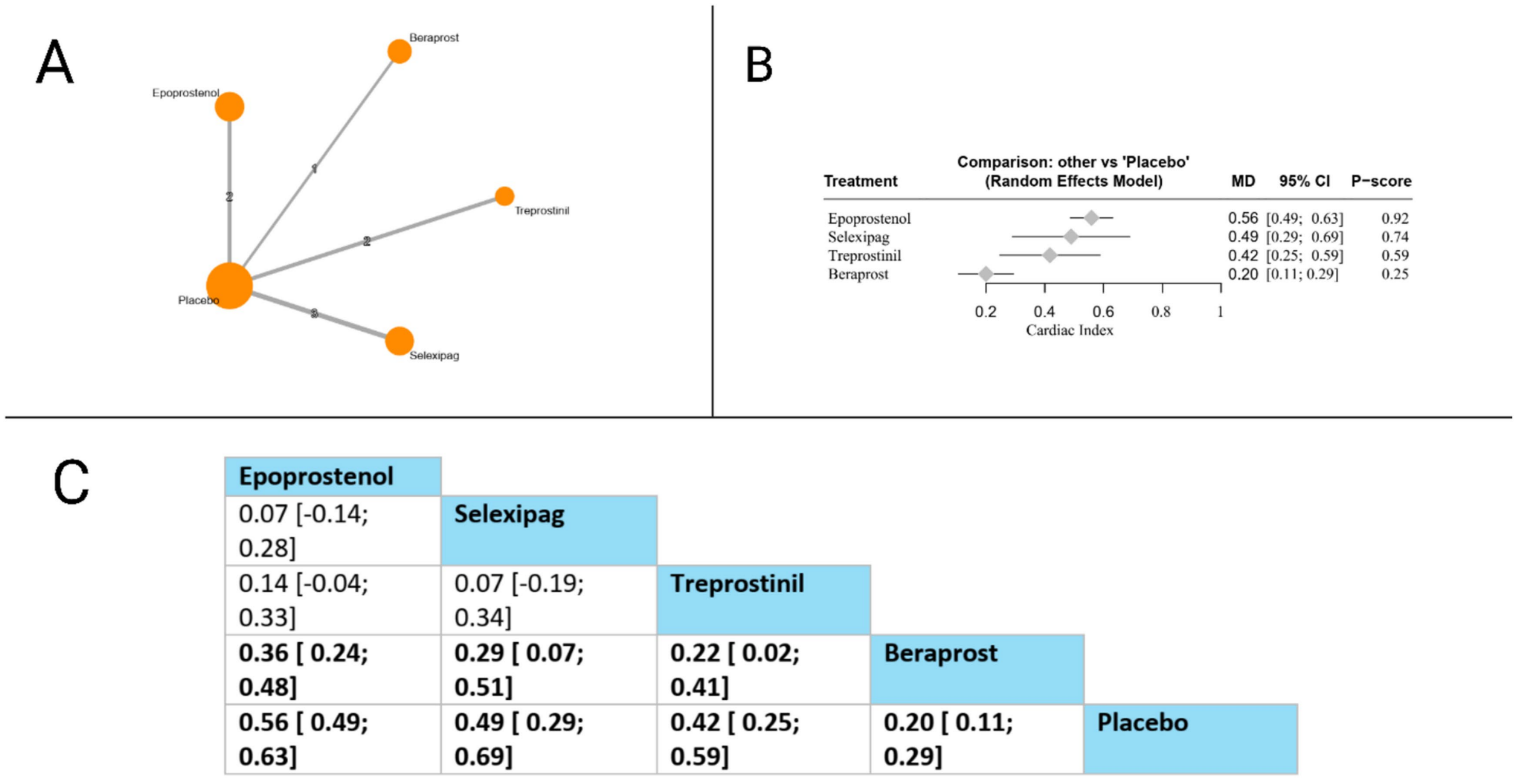
Frequentist random effect model network meta-analysis comparing cardiac index of prostanoid therapies, showing **(A)** treatment connections in network plot, **(B)** forest plot of relative risks versus placebo, and **(C)** net league matrix of pairwise comparisons with 95% confidence intervals.

Cardiac index was evaluated in eight studies. Compared to placebo, all evaluated therapies showed statistically significant improvements: epoprostenol (MD = 0.56, 95% CI: 0.49 to 0.63), selexipag (MD = 0.49, 95% CI: 0.29 to 0.69), treprostinil (MD = 0.42, 95% CI: 0.25 to 0.59), and beraprost (MD = 0.20, 95% CI: 0.11 to 0.29). In head-to-head comparisons, epoprostenol demonstrated significant superiority over beraprost (MD = 0.36, 95% CI: 0.24 to 0.48), while selexipag also showed significant advantages over beraprost (MD = 0.29, 95% CI: 0.07 to 0.51). Additionally, treprostinil was significantly more effective than beraprost (MD = 0.22, 95% CI: 0.02 to 0.41). Based on P-scores, which indicate the probability of each treatment being the most effective, epoprostenol ranked highest (P-score = 0.92), followed by selexipag (P-score = 0.74), treprostinil (P-score = 0.59), and beraprost (P-score = 0.25). These findings suggest that among prostacyclin pathway-targeting therapies, epoprostenol provides the most substantial improvement in cardiac index for patients with pulmonary arterial hypertension. The pooled studies were homogenous with *I^2^* = 43% and *X^2^-p* = 0.13 (Figure 7).

#### Clinical worsening

**Figure 8:**
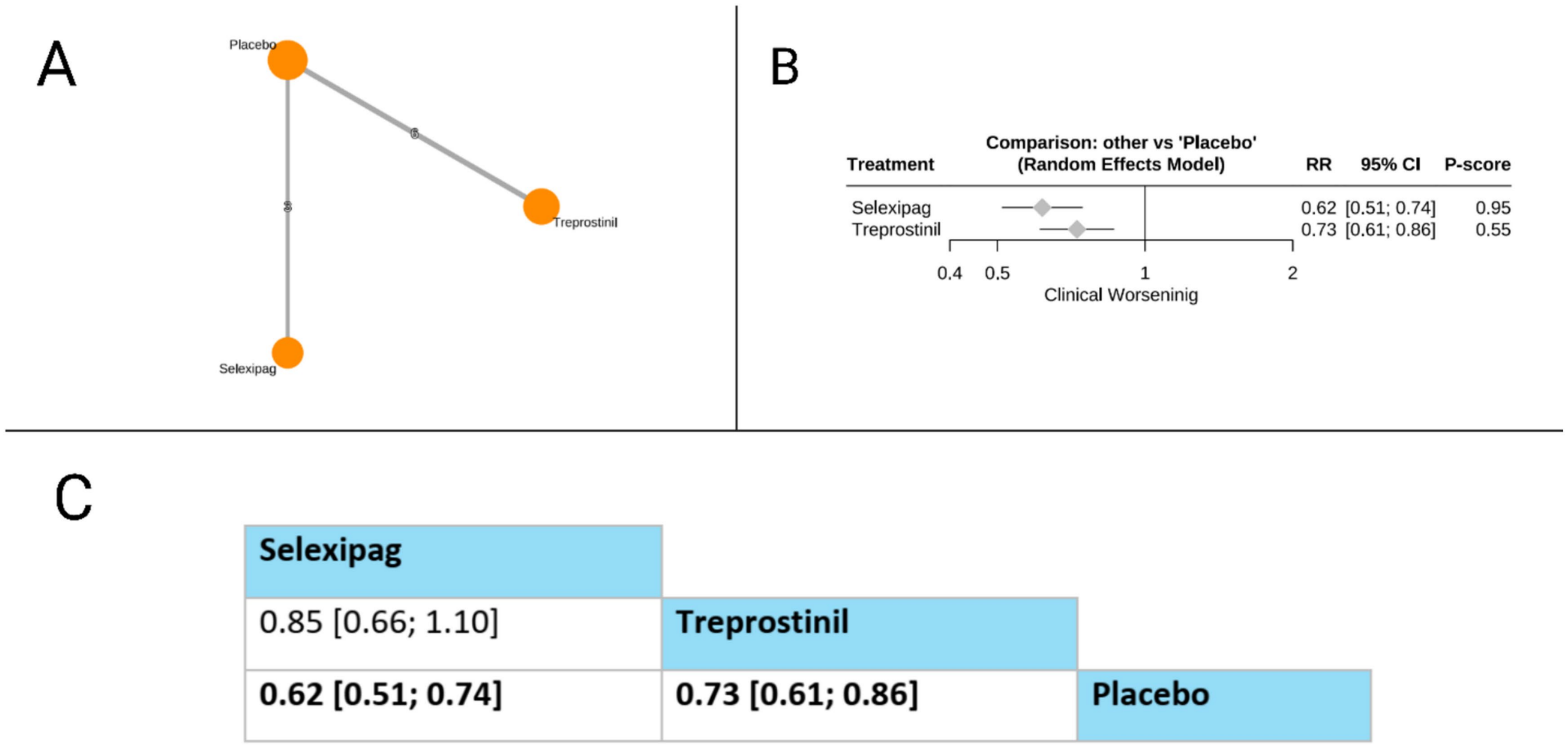
Frequentist random effect model network meta-analysis comparing clinical worsening of prostanoid therapies, showing **(A)** treatment connections in network plot, **(B)** forest plot of relative risks versus placebo, and **(C)** net league matrix of pairwise comparisons with 95% confidence intervals.

Our network showed that only two prostacyclin pathway-targeting therapies (selexipag and treprostinil) were compared for this outcome. Both selexipag (RR = 0.62, 95% CI: 0.51 to 0.74) and treprostinil (RR = 0.73, 95% CI: 0.61 to 0.86) significantly reduced the risk of clinical worsening compared to placebo. In the direct comparison between active treatments presented in the league table, selexipag showed a trend toward greater reduction in clinical worsening compared to treprostinil. However, this difference did not reach statistical significance (RR = 0.85, 95% CI: 0.66 to 1.10). Based on P-scores, which indicate the probability of each treatment being the most effective, selexipag ranked substantially higher (P-score = 0.95) than treprostinil (P-score = 0.55), suggesting that selexipag may offer the most substantial protection against clinical worsening among the evaluated prostacyclin pathway-targeting therapies for patients with pulmonary arterial hypertension. The pooled studies were homogenous with *I^2^* = 0% and *X^2^-p* = 0.97 (Figure 8).

### Secondary outcomes

The network meta-analysis of secondary outcomes revealed several significant findings. For NT-proBNP, treprostinil showed a nonsignificant reduction versus placebo (MD = −877.17, 95% CI: −1854.14 to 99.81, P-score = 0.93), while selexipag had minimal effect (MD = 23.10, 95% CI: −904.64 to 950.84). For adverse events, selexipag demonstrated significantly less cough than iloprost (RR = 0.62, 95% CI: 0.39 to 0.97) and treprostinil (RR = 0.62, 95% CI: 0.42 to 0.94), while iloprost (RR = 1.37, 95% CI: 1.05 to 1.80) and treprostinil (RR = 1.36, 95% CI: 1.14 to 1.62) significantly increased cough risk versus placebo. Regarding diarrhea, all treatments increased risk versus placebo: iloprost (RR = 0.88, 95% CI: 0.48 to 1.61), treprostinil (RR = 2.19, 95% CI: 1.72 to 2.79), and selexipag (RR = 2.43, 95% CI: 1.58 to 3.73). For jaw pain, iloprost (RR = 0.03, 95% CI: 0.0001 to 0.48), selexipag (RR = 0.04, 95% CI: 0.0001 to 0.73), and treprostinil (RR = 0.05, 95% CI: 0.001 to 0.81) all showed significantly less risk than epoprostenol. Selexipag significantly reduced hospitalization versus placebo (RR = 0.70, 95% CI: 0.55 to 0.89, P-score 0.99) and versus both iloprost (RR = 0.60, 95% CI: 0.37 to 0.98) and treprostinil (RR = 0.57, 95% CI: 0.40 to 0.80). For headache, iloprost had less risk than beraprost (RR = 0.51, 95% CI: 0.28 to 0.94) and epoprostenol (RR = 0.53, 95% CI: 0.22 to 1.29), while all treatments increased headache risk versus placebo. Regarding nausea, only treprostinil (RR = 1.69, 95% CI: 1.40 to 2.05), selexipag (RR = 1.97, 95% CI: 1.38 to 2.79), and epoprostenol (RR = 2.51, 95% CI: 1.20 to 5.26) significantly increased risk versus placebo. For syncope, treprostinil demonstrated significantly less risk than iloprost (RR = 0.11, 95% CI: 0.01 to 0.97). Treprostinil significantly increased vomiting versus placebo (RR = 2.53, 95% CI: 1.47 to 4.35) (Supplementary Figures 3–13).

## Discussion

The management of PAH has been significantly advanced by the development of therapies targeting distinct pathophysiological pathways, among which the prostacyclin pathway remains a cornerstone, particularly for patients with more severe disease or inadequate response to other therapies (6). Prostacyclin (PGI2) and its analogues exert beneficial effects through potent vasodilation, inhibition of platelet aggregation, and antiproliferative actions (9, 25). However, the available agents within this class—including epoprostenol, treprostinil (intravenous [IV], subcutaneous [SC], inhaled, oral), iloprost (inhaled), and the selective IP receptor agonist selexipag (oral)—possess distinct pharmacological properties, routes of administration, and associated clinical profiles (6, 17, 39, 41). Head-to-head comparative data are limited, complicating treatment decisions in clinical practice. This NMA, synthesizing data from 32 studies encompassing 7,819 patients, aimed to provide a comprehensive comparison of the relative efficacy and safety of prostacyclin pathway-targeting therapies across multiple critical endpoints. Treprostinil demonstrated a 34% reduction in all-cause mortality versus placebo, though epoprostenol showed a superior mortality benefit (P-score = 0.78). Functional capacity improvements were most pronounced with epoprostenol (46.84 m 6MWD vs. placebo; P-score = 0.90). Hemodynamic outcomes varied by agent: epoprostenol optimally reduced MPAP (−6.29 mmHg; P-score = 0.95) and improved right atrial pressure (−2.41 mmHg; P-score = 0.91) and cardiac index (0.56; P-score = 0.92), while iloprost showed the greatest PVR reduction (P-score = 1.00). Selexipag exhibited the strongest prevention of clinical worsening (RR = 0.62 vs. placebo; P-score = 0.95) and significantly lower hospitalization risk versus other agents. Our findings confirm the overall efficacy of this therapeutic class but highlight significant heterogeneity among agents, suggesting that treatment selection should be tailored to specific therapeutic goals and patient characteristics.

A reduction in mortality remains the ultimate goal of PAH therapy. Our NMA indicated that treprostinil significantly reduced all-cause mortality compared to placebo (RR = 0.66). This finding aligns with the long-term survival benefits observed in the FREEDOM-EV study for participants initially assigned to oral treprostinil, where an absolute risk reduction of 9% was noted over an extended follow-up period compared to those initially assigned placebo who later received open-label therapy (22, 51). Epoprostenol demonstrated the highest probability of being the most effective agent for reducing mortality (P-score = 0.78). This is consistent with its established role as the most potent prostacyclin analogue, particularly effective in high-risk patients, and supported by historical data showing improved survival compared to conventional therapy in the pre-combination era (6, 9, 11). While its invasive administration route and short half-life pose challenges, its efficacy in severe PAH remains a benchmark. Selexipag, evaluated in the GRIPHON trial, reduced a composite morbidity/mortality endpoint but did not show a statistically significant reduction in mortality alone (25). The NMA mortality signal for treprostinil and epoprostenol underscores the potent life-saving potential of targeting this pathway, especially with parenteral formulations or effective oral agents like treprostinil.

Improvement in exercise capacity, commonly measured by the 6MWD, is a key treatment target and correlates with prognosis (52–54). Epoprostenol achieved the greatest mean improvement in 6MWD versus placebo (46.84 meters) and ranked highest among the evaluated therapies (P-score = 0.90). This substantial improvement reflects its potent vasodilatory and potential positive hemodynamic effects, exceeding the typical gains observed with many oral or inhaled therapies (~20–40 meters) in pivotal trials (13, 17, 44, 55, 56). While other prostacyclin agents also improve 6MWD (6, 17, 44, 53), this analysis suggests epoprostenol offers the most robust functional benefit in terms of walk distance, though this must be balanced against its administration burden.

Hemodynamic improvement, reflecting reduced pulmonary vascular load and enhanced right ventricular (RV) function, is crucial for long-term outcomes. Epoprostenol again demonstrated superiority across several key parameters, ranking highest for reducing mean pulmonary arterial pressure (MPAP: −6.29 mmHg) and right atrial pressure (RAP: −2.41 mmHg), and for increasing cardiac index (CI: 0.56 L/min/m2). These findings align with its known potent effects on pulmonary vasodilation and potential positive effects on RV contractility or coupling. Inhaled iloprost ranked highest for PVR reduction (P-score = 1.00). While potent, inhaled therapies like iloprost primarily affect pulmonary vasculature with minimal systemic effects, potentially leading to a pronounced PVR reduction signal in NMA, though the clinical significance compared to the substantial PVR reduction also seen with epoprostenol requires careful consideration (13, 57). Significant hemodynamic improvements are crucial targets, as they correlate with RV reverse remodeling and better prognosis (6, 58, 59).

Preventing clinical worsening events and hospitalizations is a critical patient-centric outcome and a major driver of healthcare costs. Selexipag demonstrated the most favorable profile in this domain, ranking highest for preventing the composite clinical worsening endpoint (RR = 0.62 vs. placebo, P-score = 0.95). This strongly validates the primary finding of the GRIPHON trial, where selexipag significantly reduced the risk of morbidity/mortality events, primarily driven by delaying disease progression and reducing PAH-related hospitalizations (25). Furthermore, our NMA specifically found that selexipag significantly reduced hospitalization risk compared not only to placebo but also to iloprost and treprostinil. This suggests a potential advantage for selexipag in maintaining stability and reducing healthcare resource utilization, which may relate to its oral administration, pharmacokinetics, or specific IP receptor interactions. Oral treprostinil, in the FREEDOM-EV trial, also significantly reduced clinical worsening compared to placebo, primarily through delaying disease progression events, highlighting the benefit of oral prostacyclin pathway agents in modifying disease course (22).

The abstract notes distinct adverse event (AE) profiles. Prostacyclin pathway agents are known to cause dose-limiting AEs, primarily related to vasodilation (headache, flushing, nausea, diarrhea, jaw pain) (60). Parenteral therapies carry route-specific risks (infusion site pain/reactions for SC treprostinil; catheter-related bloodstream infections for IV epoprostenol/treprostinil) (6, 36, 61). Inhaled therapies are commonly associated with cough and throat irritation (6, 17, 50, 53). The choice between agents often involves balancing efficacy against the tolerability and burden associated with the specific drug and its delivery system. Selexipag’s favorable hospitalization profile might indirectly reflect better overall tolerability or adherence compared to some other agents in the NMA context, although direct comparative tolerability data remain limited. The safety profile of IV selexipag also appeared manageable in a short-term switch study, suggesting feasibility for temporary bridging (62).

### Contextualization with guidelines and clinical practice

Current international guidelines emphasize risk stratification and advocate for initial combination therapy (often ERA + PDE5i) for low- or intermediate-risk patients, escalating therapy based on treatment response (6, 63). Parenteral prostanoids, such as epoprostenol or treprostinil, are typically recommended for high-risk patients or those failing oral/inhaled combinations due to their established potency and survival benefits (6, 63). Our NMA findings broadly support this framework. Epoprostenol’s superior performance in improving hemodynamics, 6MWD, and potentially mortality reinforces its role in high-risk scenarios where maximal physiological benefit is required. The demonstrated mortality benefit of treprostinil aligns with guideline recommendations supporting its use across different risk strata, including parenteral forms for high-risk patients and oral/inhaled forms as part of combination strategies (6, 63, 64).

This analysis adds important comparative nuances. Selexipag’s robust effect on preventing clinical worsening and hospitalization positions it favorably as an add-on therapy, particularly when the goal is to maintain stability and reduce healthcare utilization, consistent with its indication and use in practice following the GRIPHON trial (6, 25, 63). The varying profiles suggest a potential for tailoring therapy: a patient requiring significant hemodynamic improvement might benefit most from epoprostenol, whereas a patient prioritizing avoidance of hospitalization might be better suited for selexipag as part of a combination regimen. The choice also depends heavily on patient preference, administration route feasibility, and tolerability.

### Strengths and limitations

This study represents, to our knowledge, the initial network meta-analysis examining prostacyclin pathway therapeutics in PAH. The primary methodological strength lies in our comprehensive evidence synthesis, incorporating data from numerous clinical investigations and substantial patient populations, thereby facilitating indirect comparative assessment across multiple prostacyclin pathway agents. This substantial dataset decreases potential type II statistical error concerns. Furthermore, our multidimensional assessment approach—examining mortality, clinical deterioration events, six-minute walk distance, hemodynamic parameters, and hospitalization rates—provides clinicians with a comprehensive therapeutic efficacy profile across critical outcome domains. This methodological approach offers distinct advantages over conventional pairwise meta-analyses by establishing relative efficacy hierarchies across the entire therapeutic class rather than isolated agent-to-agent comparisons.

Our study has several important limitations that warrant cautious interpretation. As a network meta-analysis, our findings rely heavily on indirect comparisons, which are inherently less robust than direct evidence from randomized controlled trials. The included studies exhibited substantial heterogeneity across trial designs, patient populations (including disease etiology, severity, and background therapies), endpoint definitions, and follow-up durations. Notably, we were unable to perform subgroup or sensitivity analyses stratified by treprostinil administration route (IV, oral, inhaled, SC) due to the limited number of studies per formulation, inconsistent reporting, and insufficient statistical power. This limitation is compounded by variability in dosing regimens and differences between delivery methods—such as continuous infusion (epoprostenol), inhalation (iloprost), and oral receptor agonists (selexipag)—which may confound efficacy estimates. Our necessary pooling of treprostinil data across multiple formulations may obscure route-specific effects and mask clinically relevant distinctions. Additionally, P-scores reflect the likelihood of being the most effective treatment but do not convey the magnitude or clinical significance of differences. Finally, the lack of access to individual patient data prevented more granular subgroup analyses.

### Implications and future directions

Despite these limitations, this NMA provides valuable insights for clinicians managing PAH. It reinforces the central role of the prostacyclin pathway and confirms that all agents offer clinical benefits, albeit with distinct profiles. The findings support tailoring therapy based on individual patient risk profiles, treatment goals (e.g., achieving hemodynamic targets vs. preventing clinical events), and tolerance/preference regarding administration routes. Epoprostenol’s potency remains evident, justifying its use in high-risk patients despite administration challenges. Selexipag emerges as a particularly effective agent for preventing clinical worsening and hospitalizations, supporting its use in escalation strategies. The survival benefit associated with treprostinil, particularly oral treprostinil in its trial context, is also noteworthy.

Future research should prioritize well-designed, head-to-head RCTs directly comparing different prostacyclin agents, especially across different routes of administration (e.g., oral vs. inhaled treprostinil, selexipag vs. oral treprostinil). Studies evaluating specific formulations (e.g., dry powder inhaled treprostinil) are needed. Further investigation into the long-term impact of these agents on survival, quality of life, and healthcare resource utilization, particularly in the context of modern initial dual- and triple-combination strategies, is warranted. Research focusing on specific patient phenotypes (e.g., based on comorbidities, risk scores) could help further refine personalized treatment approaches.

## Conclusion

This comprehensive NMA demonstrates that prostacyclin pathway therapies offer significant but heterogeneous benefits in PAH management. While epoprostenol exhibits superior improvements in hemodynamics and functional capacity, treprostinil reduces mortality by 34%, and selexipag excels in preventing clinical worsening and hospitalizations. These findings suggest therapy selection should be individualized based on treatment goals, patient characteristics, and administration preferences. Future research should prioritize direct head-to-head comparisons, particularly examining different administration routes of the same agent. Studies investigating long-term outcomes within modern combination therapy frameworks and research identifying optimal agents for specific patient phenotypes would further advance personalized PAH management approaches.

## Data Availability

The original contributions presented in the study are included in the article/Supplementary material, further inquiries can be directed to the corresponding author/s.
