# Supplementary material for: Comparative efficacy and safety of prostacyclin therapies for pulmonary arterial hypertension: a systematic review and network meta-analysis
Source: Front Med (Lausanne). 2025 Oct 13;12:1643220. doi: 10.3389/fmed.2025.1643220 (PMC12554579; doi:10.3389/fmed.2025.1643220)
Supplement: Supplementary file 1 [file Data_Sheet_1.docx]

**Supplementary File**

A Systematic Review and Network Meta-Analysis of Prostacyclin-Based Therapies for Pulmonary Arterial Hypertension: Comparative Efficacy and Safety

**Supplementary Table 1:** Search Strategy Appendix

| **Databases** | **Search Query** | **Filters** | **Results** |
| --- | --- | --- | --- |
| **PubMed** | ("pulmonary hypertension" OR "PAH" OR "pulmonary arterial hypertension") AND ("Epoprostenol" OR "Treprostinil" OR "Iloprost" OR "Selexipag" OR "prostacyclin analogue*" OR "prostacyclin therapy") | All | 2969 |
| **Cochrane** | ("pulmonary hypertension" OR "PAH" OR "pulmonary arterial hypertension") AND ("Epoprostenol" OR "Treprostinil" OR "Iloprost" OR "Selexipag" OR "prostacyclin analogue*" OR "prostacyclin therapy") | All | 581 |
| **Scopus** | TITLE-ABS-KEY (( "pulmonary hypertension" OR "PAH" OR "pulmonary arterial hypertension”) AND ("Epoprostenol" OR "Treprostinil" OR "Iloprost" OR "Selexipag" OR "prostacyclin analogue*" OR "prostacyclin therapy")) AND (LIMIT-TO( DOCTYPE, "ar”)) AND (LIMIT-TO ( LANGUAGE, "English”)) AND (LIMIT-TO ( EXACTKEYWORD, "Human" )) | Keywords, title, abstracts | 2820 |
| **WOS** | ("pulmonary hypertension" OR "PAH" OR "pulmonary arterial hypertension") AND ("Epoprostenol" OR "Treprostinil" OR "Iloprost" OR "Selexipag" OR "prostacyclin analogue*" OR "prostacyclin therapy") (All Fields) and Article (Document Types) and English (Languages) | All | 2401 |

**Supplementary Table 2:** Newcastle-Ottawa Scale (NOS) Assessment of Observational Studies

| **Study ID** | **Comparability:  Adjusted for: 1) Age, 2) Medical history** | **Cohort studies** | | | | | | | | |
| --- | --- | --- | --- | --- | --- | --- | --- | --- | --- | --- |
|  |  | **Selection** | | | | **Comparability** | **Outcome** | | | **Quality Score** |
|  |  | **Representativeness of the exposed cohort** | **Selection of the non-exposed cohort** | **Ascertainment of exposure** | **Demonstration that the outcome of interest was not present at the start of the study** | **Comparability of cohorts on the basis of the design or analysis** | **Assessment of outcome** | **Was the follow-up long enough for outcomes to occur** | **Adequacy of follow-up of cohorts** |  |
| **Burger 2024** |  | ***** |  | ***** | ***** | ****** | ***** | ***** | ***** | **high quality** |
| **Frantz 2015** |  | ***** |  | ***** | ***** | ****** | ***** | ***** | ***** | **high quality** |
| **McConnell 2020** |  | ***** | ***** | ***** | ***** | ***** |  | ***** | ***** | **high quality** |
| **Zamanian 2016** |  | ***** | ***** | ***** | ***** | ****** | ***** | ***** | ***** | **high quality** |
| **Nagaya 1999** |  | ***** | ***** | ***** | ***** | ***** | ***** | ***** | ***** | **high quality** |


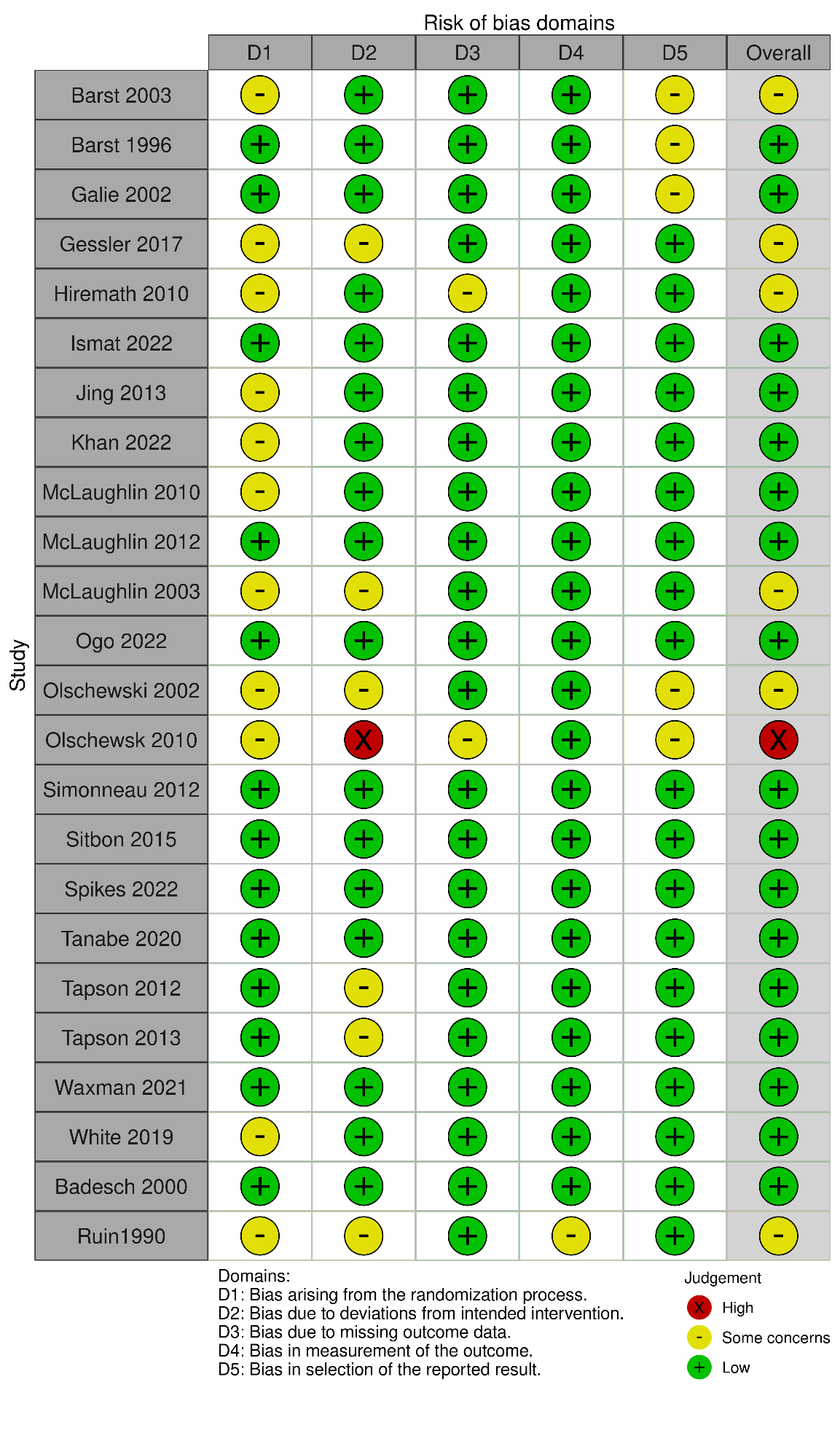


**Supplementary Figure 1**: Cochrane Risk of Bias Assessment tool 2 of Randomized Controlled Studies.


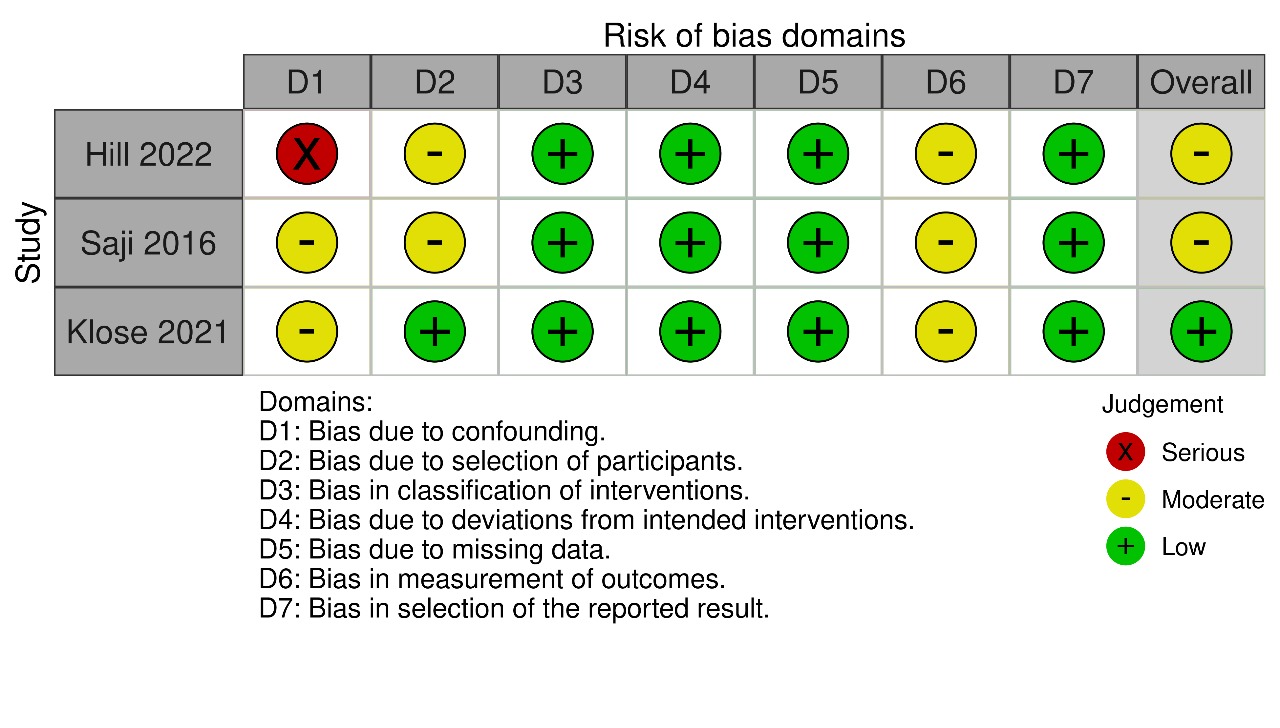


**Supplementary Figure 2**: Risk of Bias for non-randomized trials using Non-randomized Studies - of Interventions (ROBINS-1) tool.


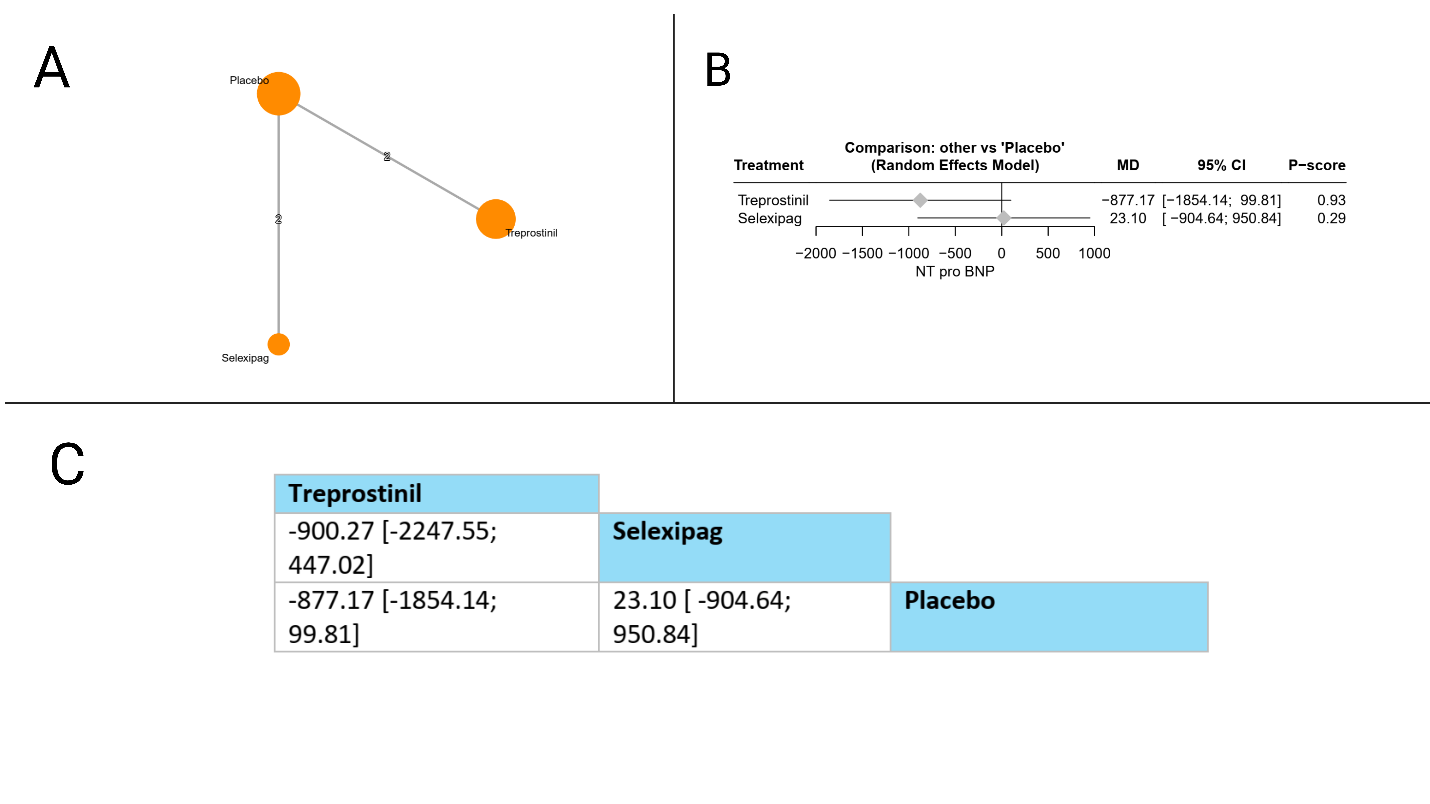


**Supplementary** **Figure 3**: Frequentist random effect model network meta-analysis comparing NT-Pro-BNP of prostanoid therapies, showing treatment connections in network plot (A), forest plot of relative risks versus placebo (B), and net league matrix of pairwise comparisons with 95% confidence intervals (C).


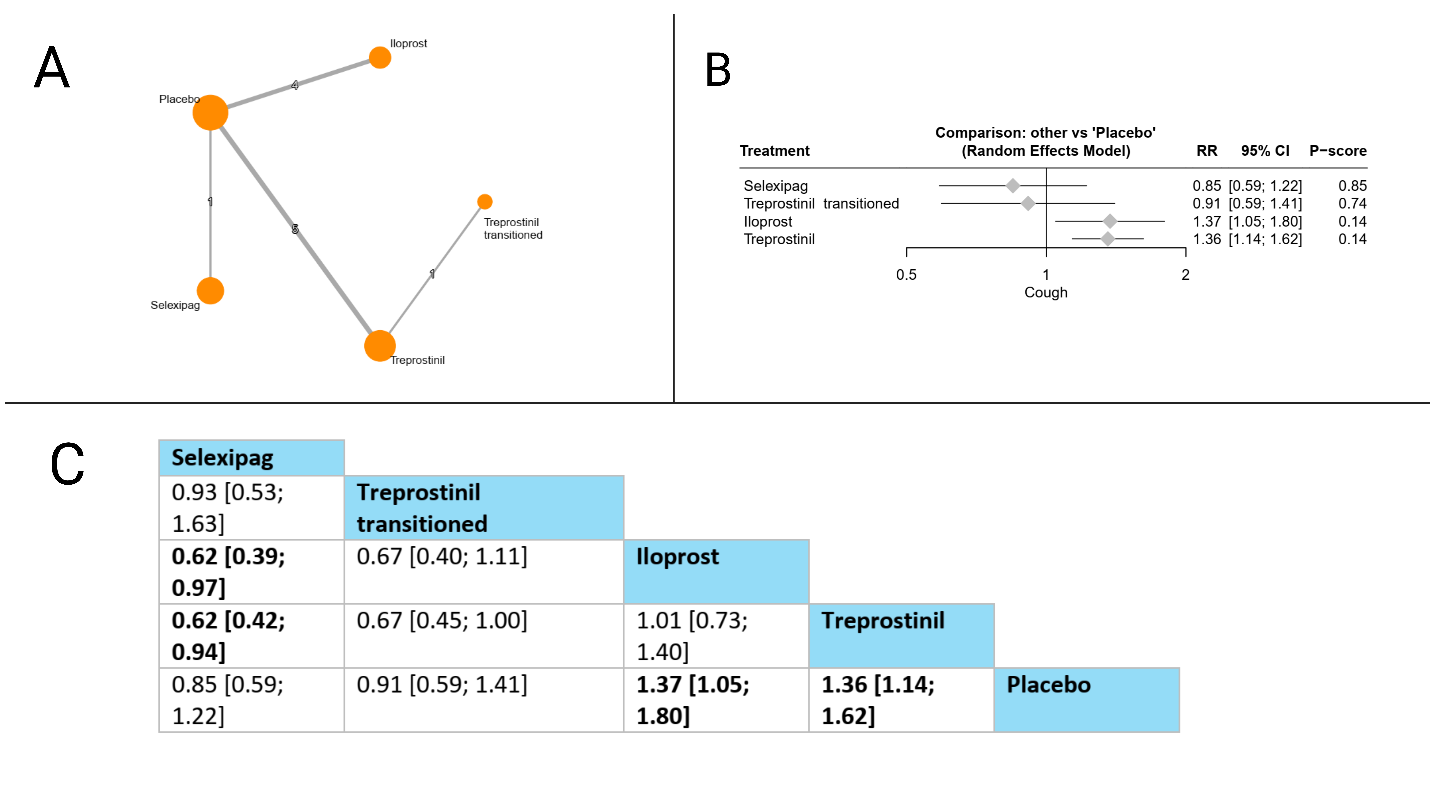


**Supplementary Figure 4**: Frequentist random effect model network meta-analysis comparing cough of prostanoid therapies, showing treatment connections in network plot (A), forest plot of relative risks versus placebo (B), and net league matrix of pairwise comparisons with 95% confidence intervals (C).


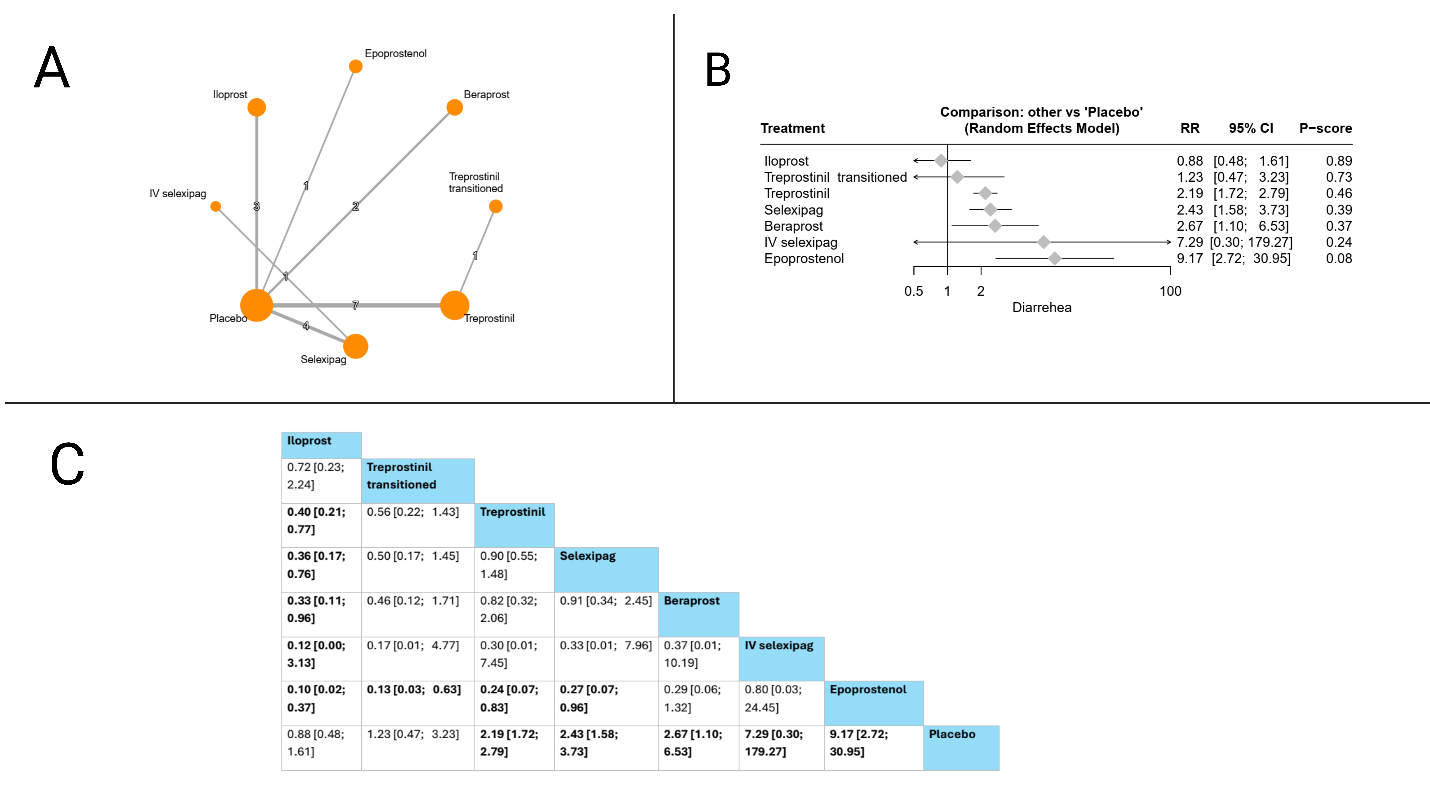


**Supplementary Figure 5:** Frequentist random effect model network meta-analysis comparing diarrhea of prostanoid therapies, showing treatment connections in network plot (A), forest plot of relative risks versus placebo (B), and net league matrix of pairwise comparisons with 95% confidence intervals (C).


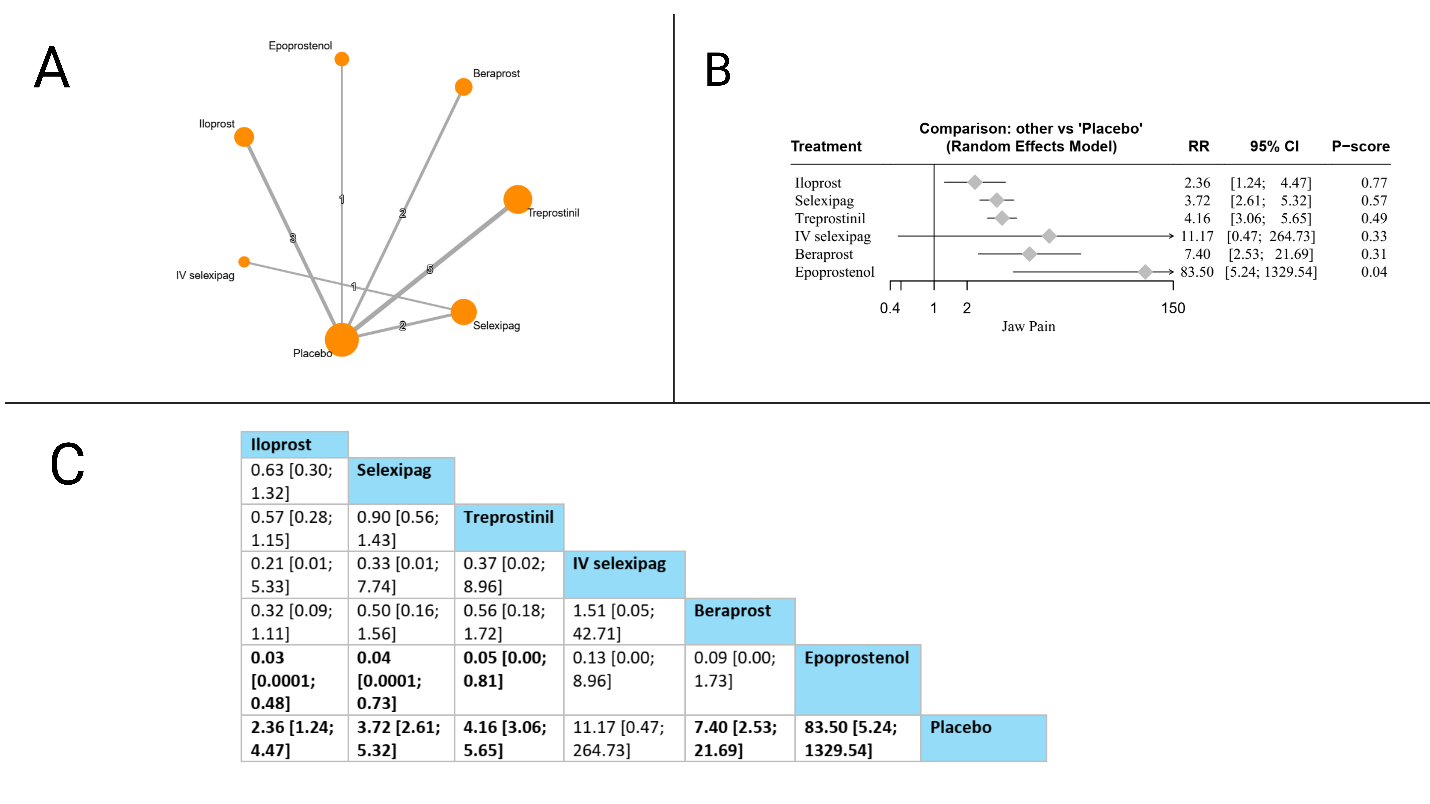


**Supplementary Figure 6**: Frequentist random effect model network meta-analysis comparing pain of prostanoid therapies, showing treatment connections in network plot (A), forest plot of relative risks versus placebo (B), and net league matrix of pairwise comparisons with 95% confidence intervals (C).


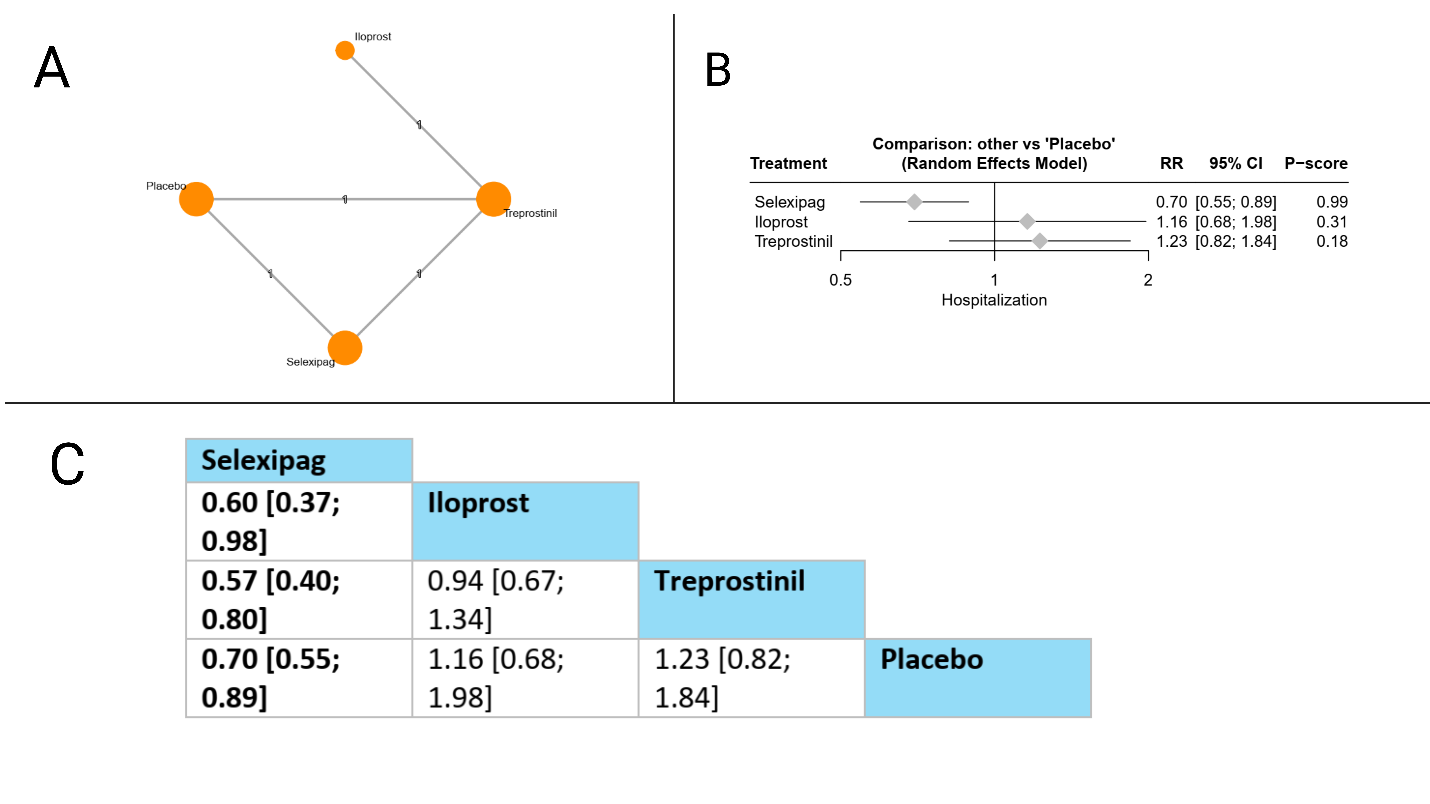


**Supplementary Figure 7:** Frequentist random effect model network meta-analysis comparing hospitalization of prostanoid therapies, showing treatment connections in network plot (A), forest plot of relative risks versus placebo (B), and net league matrix of pairwise comparisons with 95% confidence intervals (C).


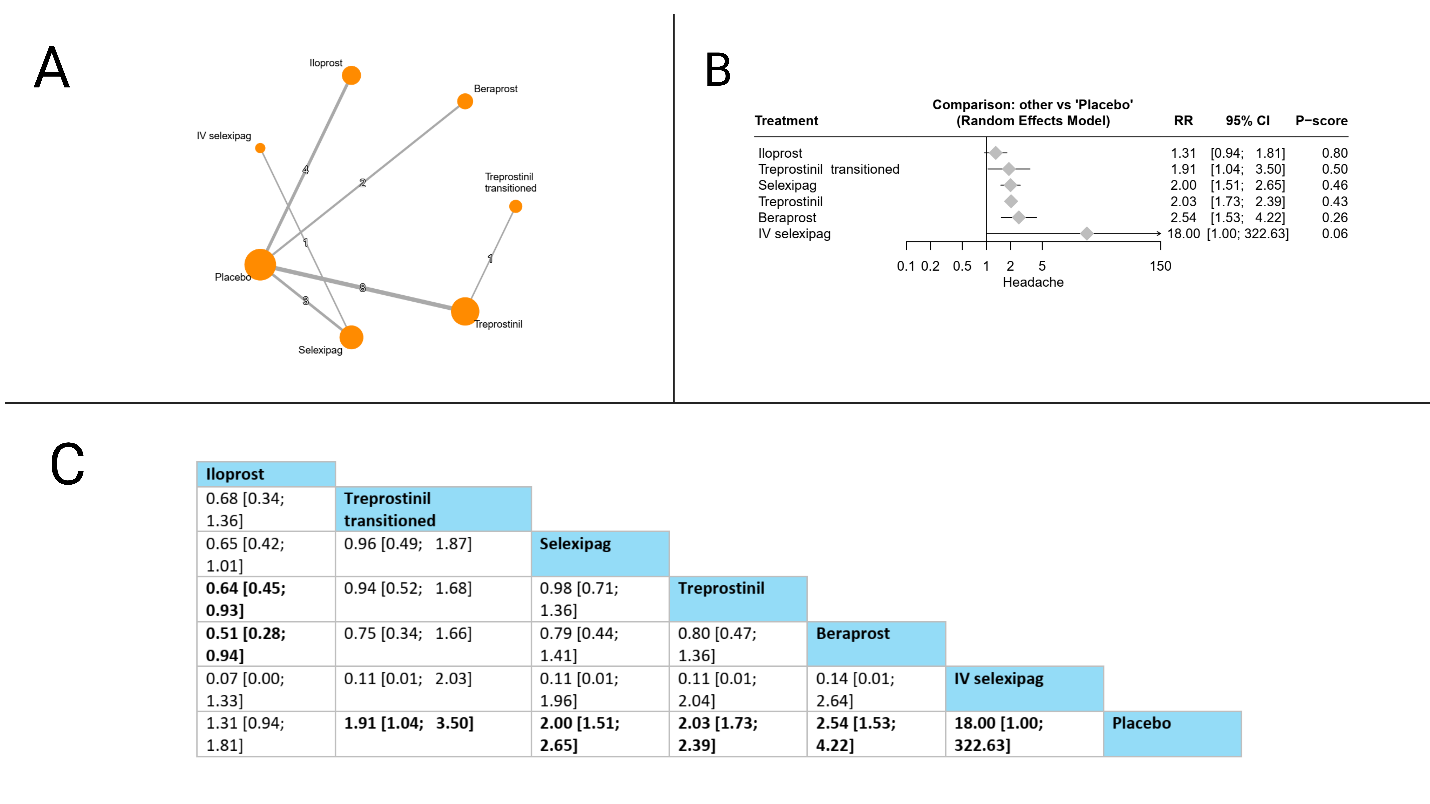


**Supplementary Figure 8:** Frequentist random effect model network meta-analysis comparing the headache of prostanoid therapies, showing treatment connections in network plot (A), forest plot of relative risks versus placebo (B), and net league matrix of pairwise comparisons with 95% confidence intervals (C).


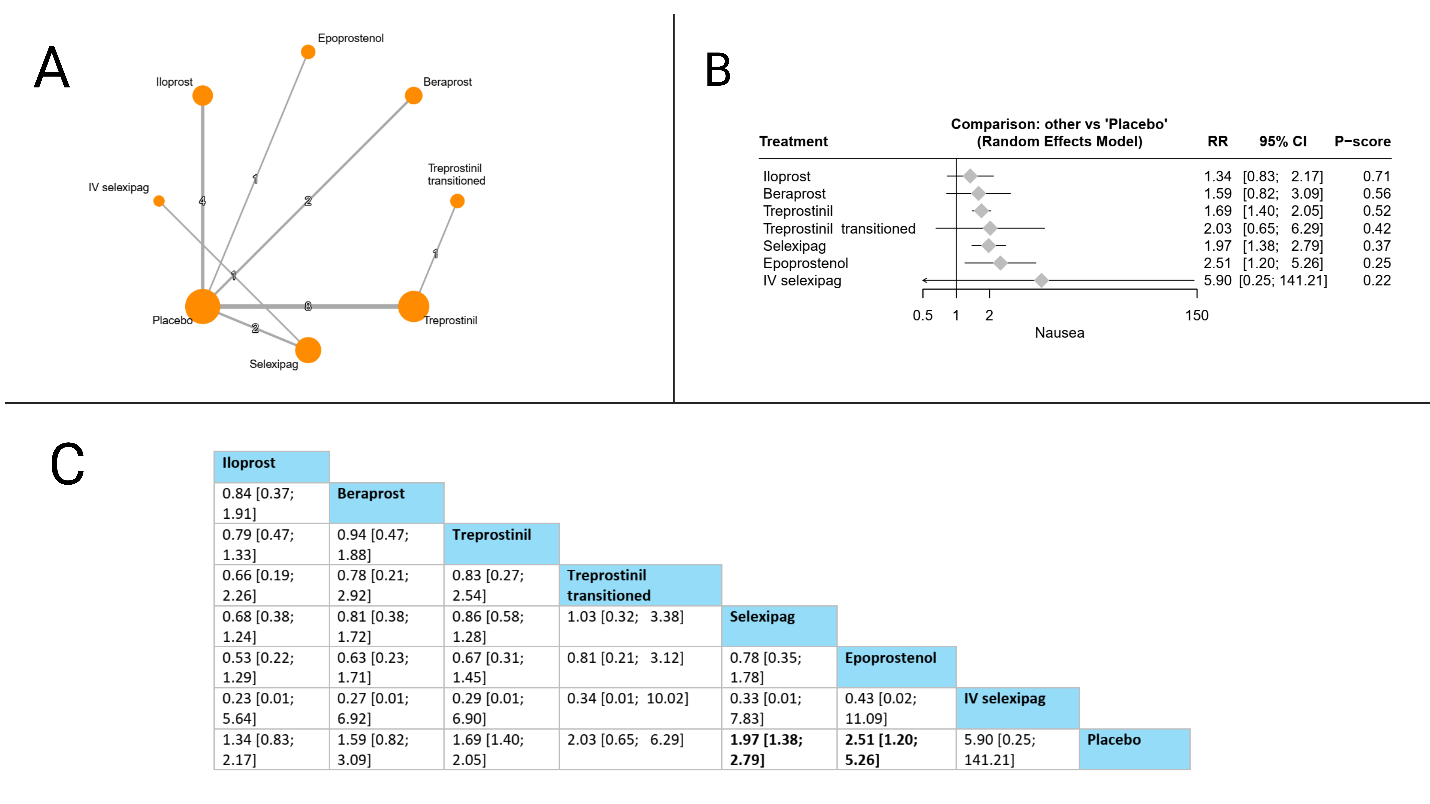


**Supplementary Figure 9**: Frequentist random effect model network meta-analysis comparing nausea of prostanoid therapies, showing treatment connections in network plot (A), forest plot of relative risks versus placebo (B), and net league matrix of pairwise comparisons with 95% confidence intervals (C).


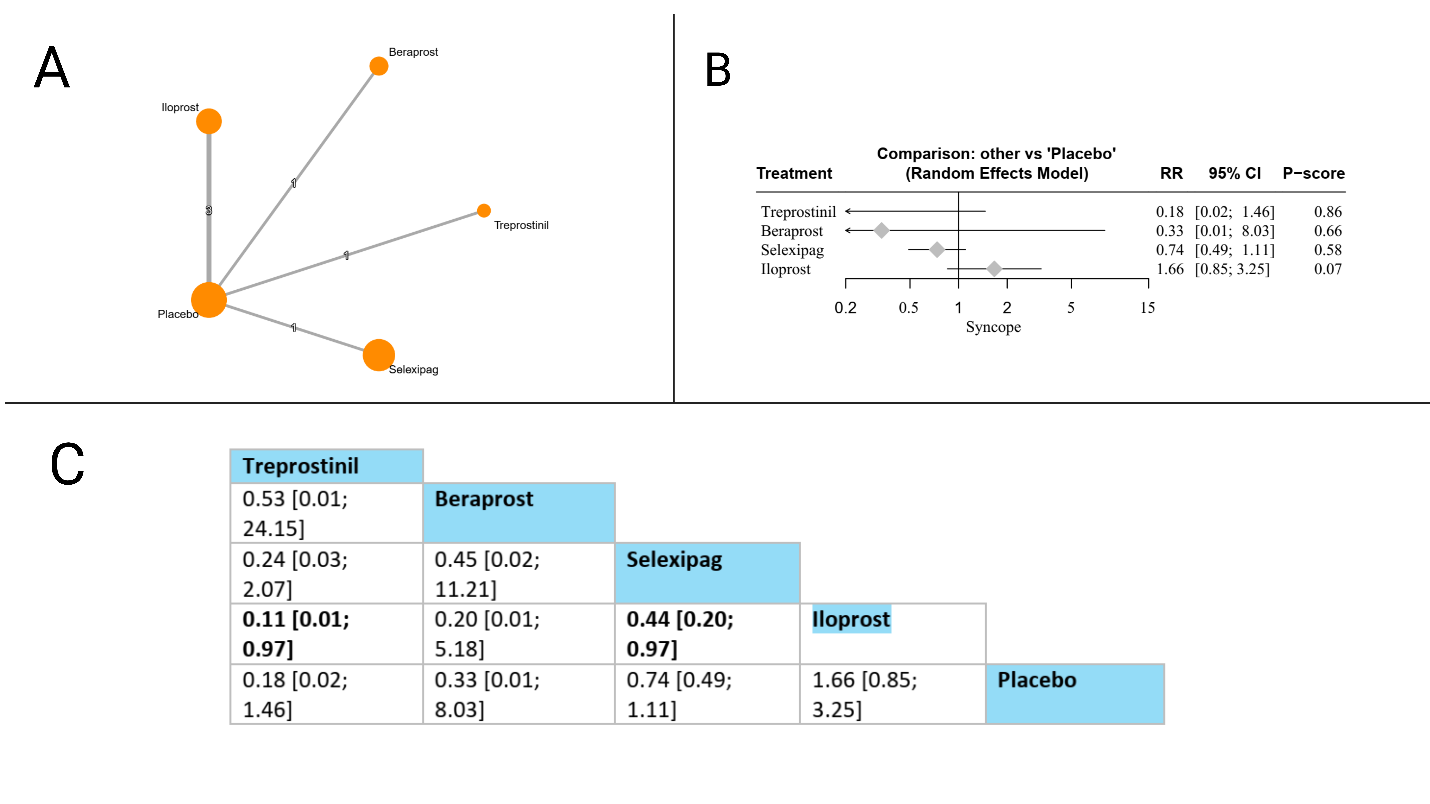


**Supplementary Figure 10**: Frequentist random effect model network meta-analysis comparing syncope of prostanoid therapies, showing treatment connections in network plot (A), forest plot of relative risks versus placebo (B), and net league matrix of pairwise comparisons with 95% confidence intervals (C).


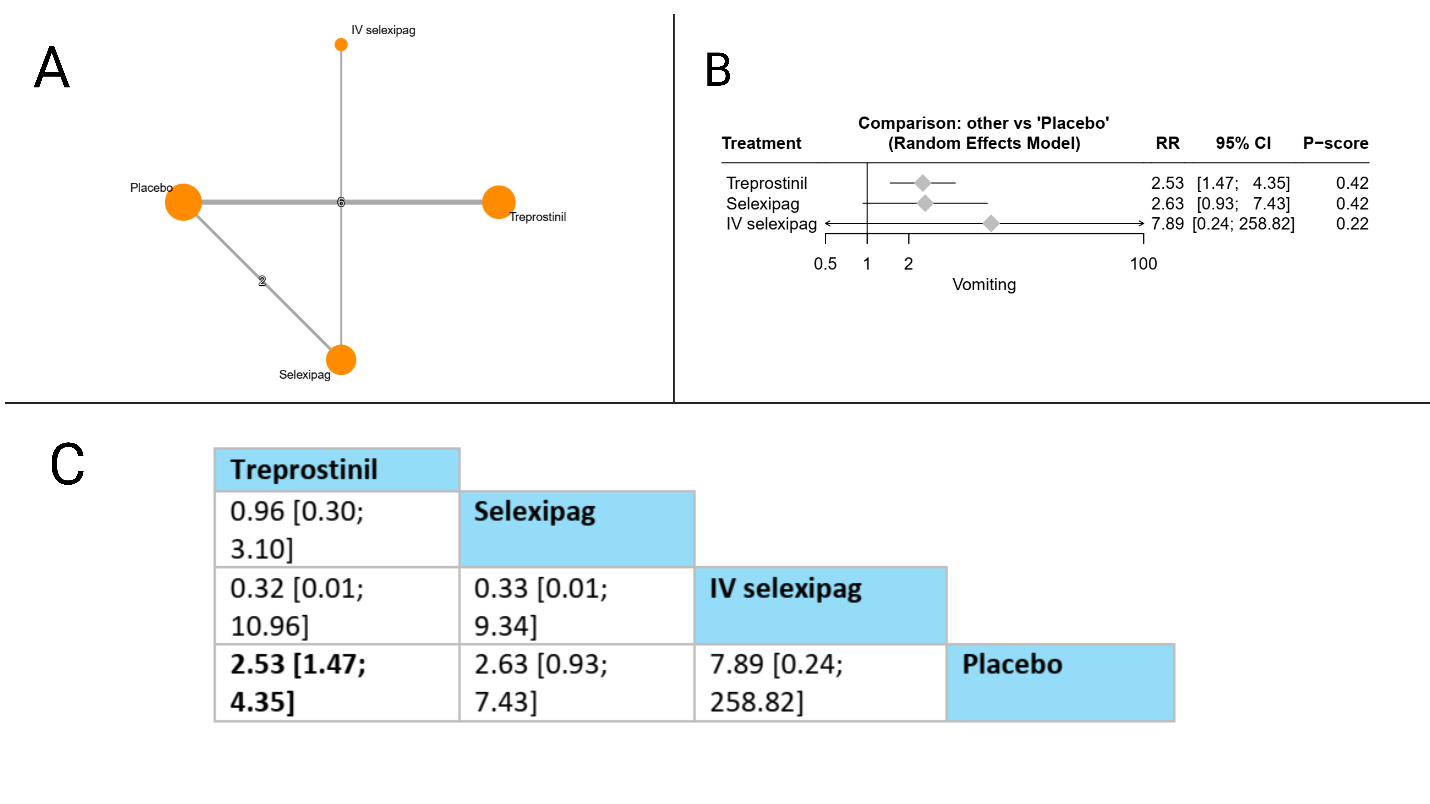


**Supplementary Figure 11**: Frequentist random effect model network meta-analysis comparing vomiting of prostanoid therapies, showing treatment connections in network plot (A), forest plot of relative risks versus placebo (B), and net league matrix of pairwise comparisons with 95% confidence intervals (C).


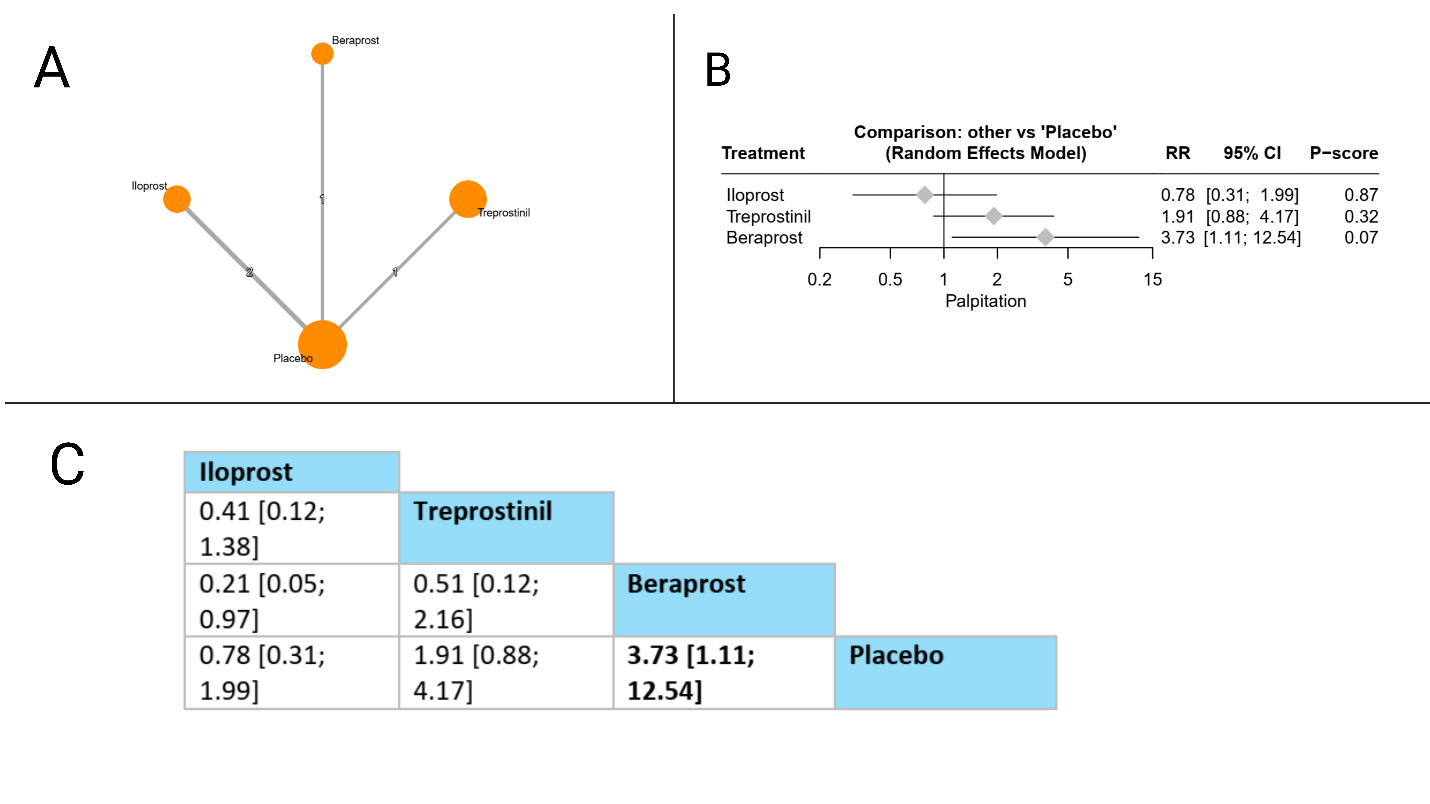


**Supplementary Figure 12:** Frequentist random effect model network meta-analysis comparing palpitation of prostanoid therapies, showing treatment connections in network plot (A), forest plot of relative risks versus placebo (B), and net league matrix of pairwise comparisons with 95% confidence intervals (C).


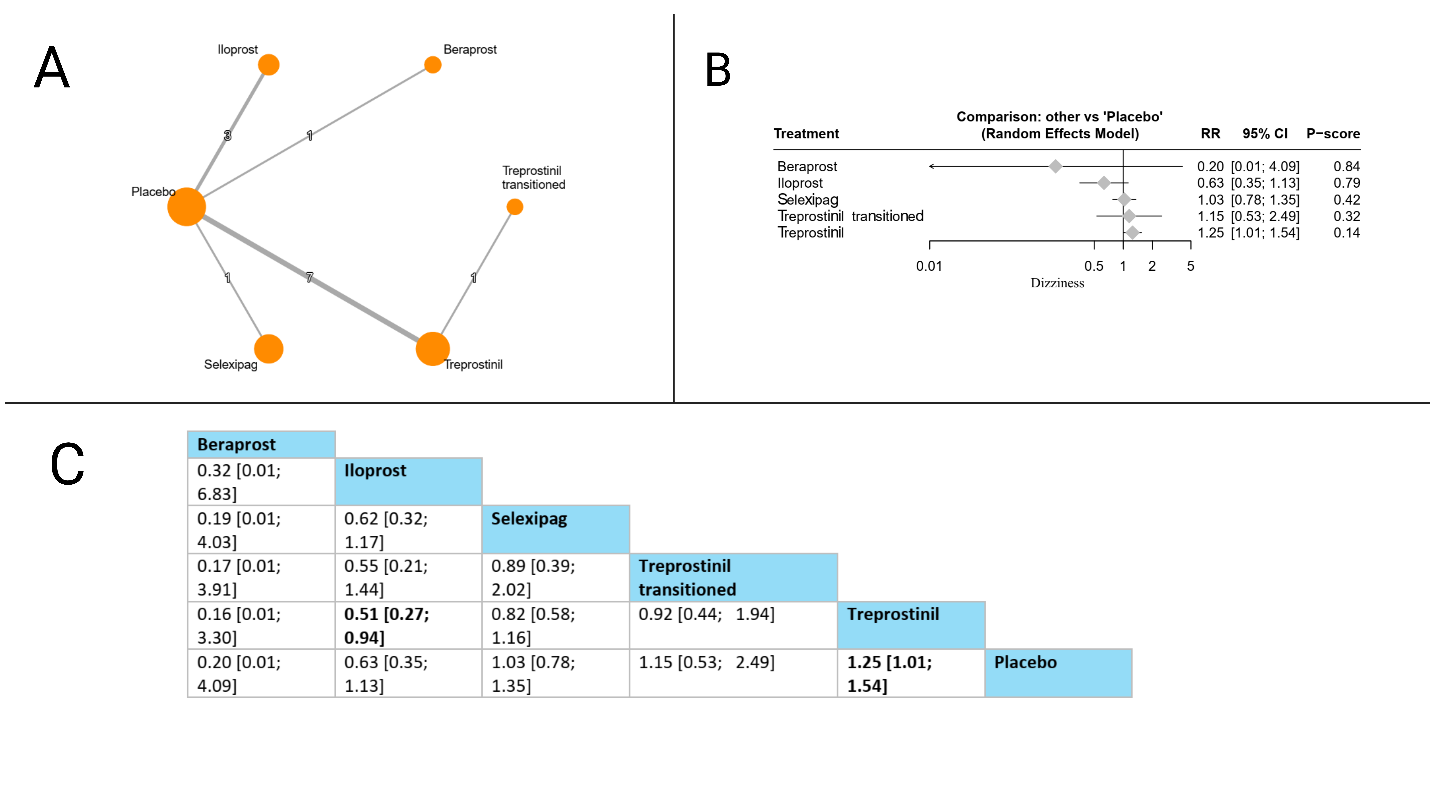


**Supplementary Figure 13**: Frequentist random effect model network meta-analysis comparing dizziness of prostanoid therapies, showing treatment connections in network plot (A), forest plot of relative risks versus placebo (B), and net league matrix of pairwise comparisons with 95% confidence intervals (C).
